# Lipidation of Naturally Occurring α-Helical Antimicrobial Peptides as a Promising Strategy for Drug Design

**DOI:** 10.3390/ijms24043951

**Published:** 2023-02-16

**Authors:** Marta Makowska, Paulina Kosikowska-Adamus, Magdalena Zdrowowicz, Dariusz Wyrzykowski, Adam Prahl, Emilia Sikorska

**Affiliations:** 1 Department of Organic Chemistry, Faculty of Chemistry, University of Gdansk, Wita Stwosza 63, 80-308 Gdansk, Poland; 2 Department of Physical Chemistry, Faculty of Chemistry, University of Gdansk, Wita Stwosza 63, 80-308 Gdansk, Poland; 3 Department of General and Inorganic Chemistry, Faculty of Chemistry, University of Gdansk, Wita Stwosza 63, 80-308 Gdansk, Poland

**Keywords:** antimicrobial peptides, lipopeptides, structure-activity relationship, self-assembly, AMP activity improvement

## Abstract

In this paper, we describe the chemical synthesis, preliminary evaluation of antimicrobial properties and mechanisms of action of a novel group of lipidated derivatives of three naturally occurring α-helical antimicrobial peptides, LL-I (VNWKKVLGKIIKVAK-NH_2_), LK6 (IKKILSKILLKKL-NH_2_), ATRA-1 (KRFKKFFKKLK-NH_2_). The obtained results showed that biological properties of the final compounds were defined both by the length of the fatty acid and by the structural and physico-chemical properties of the initial peptide. We consider C_8_–C_12_ length of the hydrocarbon chain as the optimal for antimicrobial activity improvement. However, the most active analogues exerted relatively high cytotoxicity toward keratinocytes, with the exception of the ATRA-1 derivatives, which had a higher selectivity for microbial cells. The ATRA-1 derivatives had relatively low cytotoxicity against healthy human keratinocytes but high cytotoxicity against human breast cancer cells. Taking into account that ATRA-1 analogues carry the highest positive net charge, it can be assumed that this feature contributes to cell selectivity. As expected, the studied lipopeptides showed a strong tendency to self-assembly into fibrils and/or elongated and spherical micelles, with the least cytotoxic ATRA-1 derivatives forming apparently smaller assemblies. The results of the study also confirmed that the bacterial cell membrane is the target for the studied compounds.

## 1. Introduction

According to the report of the World Health Organization (WHO), the uprising resistance of pathogenic bacterial strains to commonly used antimicrobial drugs is claimed as one of the main global problems threatening public health and the economy [1]. Taking into account that antibiotic resistance is a natural phenomenon, it seems highly plausible that every subsequent antibiotic approved for treatment can induce, sooner or later, the emergence of resistant strains [2]. However, the misuse and/or abuse of antibiotics as well as inadequate sanitation and lack of effective prevention of infections accelerate the spread of microorganisms and the emergence of resistance to antibiotics [1]. Pathogenic microorganisms evade the effects of antibiotics by one of four mechanisms: limitation of drug uptake, modifying drug target, drug inactivation and active drug efflux [3].

Antimicrobial peptides (AMPs), due to their important role in the innate immunity of all living organisms, could serve as highly promising solution to the problem of drug resistance of pathogenic microorganisms [4,5]. A wide range of antimicrobial activity of AMPs against bacteria, viruses, fungi, and protozoa [6] predefines their potential use in the design of novel antibiotic therapies. Moreover, AMPs have additional advantages such as anti-inflammatory, wound-healing and anticancer properties [7]. 

AMPs are a diverse group of compounds, but they share some common structural and physicochemical features that determine their activity, such as [5,7]: conformation, net charge, and amphipathicity. In terms of conformation, AMPs can be divided into four groups: α-helical, β-sheets, cyclic and peptides with extended loop structures [8,9]. Linear α-helical peptides are the most abundant in nature and are the most extensively studied in terms of structure–activity relationships (SAR) [10]. Another important parameter characteristic of most of AMPs is cationicity, which varies even within one family of peptides. It was found that the optimal net charge is in the range of +4 to +6 [10], which guarantees the electrostatic interactions with negatively charged membranes of microorganisms [8,9,10,11]. Finally, the presence of nonpolar amino acids in the peptide sequence ensures interactions of AMPs with the hydrophobic core of bacterial membranes [9]. It is assumed that the percentage of hydrophobic residues should be in the range of 40–60% to maintain the amphipathic structure of the peptide [10]. Thus, the amphipathic structure and the overall positive charge of AMPs determine their mode of action. Cationic AMPs are attracted to the negatively charged bacterial cell membranes and interrupt their structures according to the barrel stave, carpet or toroidal models [12,13]. Besides membranolytic activity, some AMPs can also penetrate the cell membranes in a non-invasive way and interact with internal molecular targets, inhibiting the biosynthesis of the membrane components [5] or affecting the process of the replication of the genetic material of the microorganisms [14,15,16]. Moreover, AMPs can regulate the levels of cytokine release, neutralize bacterial toxins and activate T lymphocytes [17]. This diversity of actions makes these compounds less prone to induce the resistance of microorganisms [6]. 

Despite high therapeutic potential of AMPs, their use as medical agents is still a big challenge. The key limitations of AMPs result from their low stability in the physiological environment, which minimizes their efficient delivery to the target site. Among other restrictive factors of AMPs are their salt sensitivity, high cytotoxicity and immunogenicity toward human tissues [18]. However, there is a wide group of chemical modifications that improve the biological and physicochemical properties of antimicrobial peptides, including cyclization, PEGylation, phosphorylation, lipidation and introduction of D-amino acids into peptide sequences [19]. Among the aforementioned procedures, lipidation is one of the most important naturally occurring post-translational modifications, which regulates the functions of peptides and proteins and increases their affinity for cell membranes as well [19,20]. Lipidation can have a beneficial effect on the antimicrobial activity of AMPs by enhancing their hydrophobic interactions with bacterial membranes [21]. It also affects the solubility, tendency to self-assembly, cytotoxicity and selectivity of compounds [20], which strictly depend on the length, degree of saturation or branching of the fatty acid chain attached to the peptide [22]. The self-assembly of antimicrobial peptides is one of the key features that determines the mechanism of their biological activity. The knowledge of whether nanostructures formed by AMPs break up into monomers when incorporated into the membrane or if peptides co-assemble with lipids into new structures is crucial for understanding their mode of action. The latter could also shed a light on the capability of AMPs to penetrate the peptidoglycan and lipopolysaccharide layers of Gram-positive and Gram-negative bacteria, respectively. Self-assembly often results in increased stability and proteolytic resistance of antimicrobial peptides [12], which leads to a higher concentration of AMPs in the target sites and consequently to more efficient elimination of pathogens [13]. Moreover, it may have an impact on peptide selectivity by reducing the effective peptide hydrophobicity and thus the affinity toward membranes composed of zwitterionic lipids (such as the outer layer of healthy eukaryotic cell membranes) [23]. Finally, nanostructures formed by AMPs are promising tools for the efficient transport of drugs across the bacterial membrane, which can be used in targeted therapy due to the possibility of entrapping medicines inside them [23]. On the other hand, the self-assembly process might compete with the interactions of AMPs with microbial membranes and thus leads to the reduction of their antimicrobial activity [24]. Previous studies have proven that the attachment of fatty acids to the N-terminus of peptides increases their activity against multidrug-resistant pathogens. However, the unbalanced extension of the hydrocarbon chain results in stronger interactions inside the formed aggregates and in the loss of antimicrobial properties [21,24]. The elongation of the fatty acid hydrocarbon chain is also correlated with the enhanced cytotoxicity of synthesized compounds against healthy human cells [24]. 

In the present work, we have studied the effect of lipidation on the antimicrobial properties of three naturally occurring α-helical peptides [25,26,27,28]: LL-I (VNWKKVLGKIIKVAK-NH_2_), LK6 (IKKILSKIKKLLK-NH_2_), and ATRA-1 (KRFKKFFKKLK-NH_2_) (Table S1, Supplementary Material). The selected peptides fulfilled the following criteria: short amino acid sequence (up to 15 amino acids), high content of L-lysine residues, positive net charge, helicity, and confirmed activity against a wide range of microorganisms. Within the scope of our studies, we analyzed cytotoxicity, conformation and self-assembly properties of the most promising lipopeptides. In addition, we studied the interactions of the selected lipopeptides with artificial membrane models and visualized their effect on *S. aureus* cells. The performed studies allowed for a thorough analysis of the effect of lipidation on the structure–activity relationship.

## 2. Results and Discussions

### 2.1. Antimicrobial Activity Assays

Minimum inhibitory concentration (MIC) values against the representative strains of Gram-positive and Gram-negative bacteria, as well as fungi *Candida* spps, are presented in Table 1. As can be seen, the lipopeptides were active against Gram-positive bacteria and fungi, while Gram-negative *E. coli* was the most resistant strain. The antimicrobial properties were strongly dependent on the type of the attached fatty acid. The best activity with MIC values of 3.13–12.5 µg/mL against Gram-positive bacteria was observed for derivatives with hydrocarbon chain that comprised C_8_–C_12_ carbon atoms. The extension of the carbon chain length with the consequent increase in the lipopeptide hydrophobicity led to reduction of the activity against representative Gram-positive bacterial strains but improved eradication of fungi strains, which is in a good agreement with the previous studies [29,30]. Nevertheless, in the case of palmitoyl-modified LL-I and LK6 analogues, a marked drop in antifungal activity was noted, while C_16_-ATRA-1 still remained active with MIC not exceeding 3.13 µg/mL. This discrepancy probably resulted from the highest overall positive charge of the ATRA-1 analogues. Vieira and Carmona-Ribeiro [31] showed that the critical step determining the antifungal effect of cationic compounds is the reversion of the cell surface charge from negative to positive rather than cell lysis, which well correlates with our results. Moreover, the highest overall charge decreased the ability of the peptide to self-assembly or facilitated dissociation of the monomers from assemblies during membrane association. Taking into account that the fungal envelope is more resistant to penetration by AMPs in comparison to the bacterial one, lipopeptide oligomerization could have a negative impact on antifungal properties, as it may impair the penetration ability of the lipopeptide through the cell wall and thus prevent interaction with the cell membrane [30].

Detailed analysis of the relationship between the optical density at 600 nm and the concentration of lipopeptides showed a surprising decrease in the antimicrobial activity in the concentration range exceeding the determined MIC values (Figure S1, Supplementary Material). In a typical relationship, the pathogen’s number decreases in a concentration-dependent manner over a whole concentration range. However, in the case of the studied lipopeptides, the observed anomaly arose from the lipopeptides’ self-assembly, which reduced their affinity to the lipid bilayer of microorganisms [32].

### 2.2. Evaluation of Cytotoxicity

The results of the cytotoxicity of the most active lipopeptides against the immortal human keratinocytes cell line (HaCaT), human breast cancer cells (MCF-7 line, ATCC) and human prostate cancer cells (PC3 line, ATCC) are shown in Figure S2 (Supplementary Material) and Table 2. As can be seen, the compounds C_8_-ATRA-1 and C_10_-ATRA-1 are characterized by the lowest cytotoxicity to human keratinocytes. An increase in the length of the hydrocarbon fatty acid chain by two methylene groups reduced the cytotoxicity ca. 1.5 times. Both ATRA-1 conjugates showed high cytotoxic activity against the MCF-7 cell line, making them a promising candidate in anti-cancer drug development. The C_10_-ATRA-1 seems to be the most attractive, as its selectivity indexes are 9.47 (MCF-7) and 3.92 (PC3). This means that an almost 9.5-times lower lipopeptide concentration is required to inhibit the biological processes of MCF-7 cells and an almost 4-times lower concentration in the case of PC3 compared to healthy human cells. A similar tendency is observed for C_8_-ATRA-1. Unfortunately, in the remaining cases, lipidation gave a high cytotoxicity to both healthy and neoplastic cell lines.

### 2.3. Characterization of Peptide Assemblies

**Figure 1:**
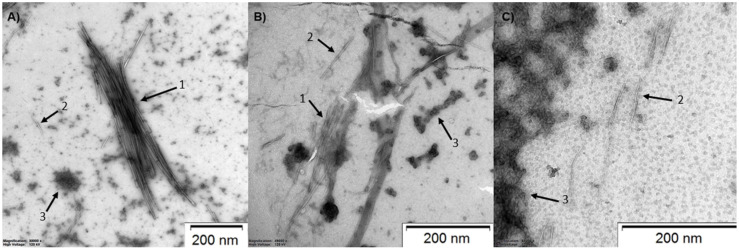
TEM images of (**A**) C_8_-LL-I, (**B**) C_8_-LK6, (**C**) C_10_-ATRA-1; 1—fibrils, 2—cylindrical micelles, 3—spherical or blocks of spherical micelles. Each peptide at a concentration of 2 mg/mL was incubated in the PBS buffer at 37 °C by 24 h.

To examine a size distribution (hydrodynamic diameter) of oligomeric species of the selected lipopeptides, C_8_-LL-I, C_8_-LK6 and C_10_-ATRA-1, in water and phosphate-buffered saline (PBS, pH 7.4), dynamic light scattering (DLS) was performed (Figure S3, Supplementary Material). The measurements were carried out for various concentrations of the lipopeptides (0.5–2 and 0.25–2 mM for salt-free and PBS solutions, respectively). The DLS results demonstrated the existence of multiple populations of self-assembled oligomeric species for the studied lipopeptides within the applied concentration range. In general, the average particle size distributions did not alter significantly with dilution. However, a decrease in concentration promoted the narrowing size distribution of nanostructures in most cases, except for C_10_-ATRA-1 in PBS. In 0.5 mM salt-free aqueous solution, the highest scattering intensity was centered at 404 ± 37, 327 ± 102 and 284 ± 105 nm for C_8_-LL-I, C_8_-LK6 and C_10_-ATRA-1, respectively. Transfer of the lipopeptides to the PBS resulted in a drastic shift of the peaks with the highest scattering intensity to larger values of the hydrodynamic diameters, 3360 ± 165, 2596 ± 512 and 2808 ± 1193 nm for 0.25 mM C_8_-LL, C_8_-LK6 and C_10_-ATRA-1, respectively. For comparison, the hydrodynamic diameters of the described compounds in 2 mM solutions in the PBS were found at 3844 ± 1031, 3047 ± 750 and 1040 ± 343 nm, respectively. Despite the apparent monodisperse distribution observed for C_8_-LL-I and C_8_-LK6, the spread of the hydrodynamic diameters reflected in the standard deviations is rather wide, indicating the inhomogeneous and fluctuating distribution of peptide assembly sizes. In the PBS solution, an increase in ionic strength partially screened the positive charges on the peptide molecules, reducing the electrostatic repulsions between them, and consequently facilitating the self-assembly. However, such large hydrodynamic diameters might also result from lateral association of lipopeptide assemblies and mutual diffusion. Therefore, to visualize the assemblies, the lipopeptides were examined with transmission electron microscopy (TEM) after 24 h incubation in the PBS solution. The TEM provided evidence for the formation of clumped and isolated fibrils, blocks of cylindrical micelles and spherical micelles, as shown in Figure 1.

### 2.4. Conformational Analysis

**Figure 2:**
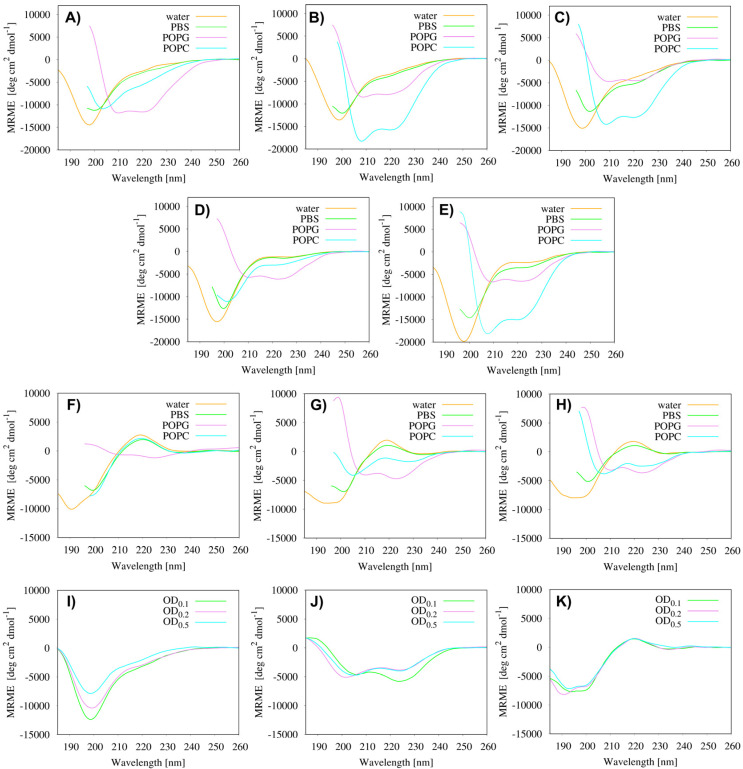
Far-UV CD spectra of (**A**) LL-I, (**B**) C_8_-LL-I, (**C**) C_10_-LL-I, (**D**) LK6, (**E**) C_8_-LK6, (**F**) ATRA-1, (**G**) C_8_-ATRA-1 and (**H**) C_10_-ATRA-1 recorded in water, PBS, POPC and POPG. Far-UV CD spectra of (**I**) C_8_-LL-I, (**J**) C_8_-LK6, (**K**) C_10_-ATRA-1 recorded in the presence of *S. aureus*. MRME denotes mean residue molar ellipticity.

The CD spectra were recorded for the parent peptides and the most active lipopeptides in water, PBS and liposomes, POPC (1-palmitoyl-2-oleoyl-*sn*-glycero-3-phosphocholine) and POPG (1-palmitoyl-2-oleoyl-*sn*-glycero-3-[phospho-rac-(1-glycerol)]) (Figure 2). The zwitterionic POPC liposomes imitated eukaryotic membranes, while POPG liposomes with their anionic character imitated negatively charged microbial membranes [33]. In water and PBS, all the tested compounds adopted an unordered conformation. The addition of POPG or POPC lipids induced the formation of an α-helical structure with characteristic minima at 208 and 222 nm in the CD curves, except for ATRA-1 in POPC. As can be seen, lipidation induced visible increases in the helical content in the presence of the zwitterionic POPC phospholipids. Moreover, in the case of the LL-I and LK6 derivatives, a clearly higher helicity was observed in the POPC (40–48%) vesicles than in the POPG ones (19–27%). In turn, in the case of ATRA-1 derivatives, the opposite tendency was noted (Table 3), which correlated well with their lowest cytotoxicity. Moreover, the θ_222_/θ_208_ ratio ≥ 1.0 determined for the parent LK6 and ATRA-1 peptides, and C_8_-LK6, C_8_-ATRA-1 and C_10_-ATRA-1 derivatives in the POPG, indicated the existence of a coiled-coil motif, which probably resulted from the formation of stable peptides’ assemblies, while the θ_222_/θ_208_ ratio ≤ 0.88 in the POPC for all studied lipopeptides suggested the presence of isolated α-helices [34,35,36].

Additionally, the conformational preferences of the selected lipopeptides, C_8_-LK6, C_8_-LL-I and C_10_-ATRA-1, were determined in the presence of *S. aureus* with various bacterial cell count of the inoculum (Figure 2). The selection of *S. aureus* cells was determined by the results of antimicrobial assays, which clearly showed that this bacterial strain is the primary target for the tested compounds. Unexpectedly, the CD spectra indicated that C_8_-LK6 was the only one that adopted the α-helical structure (17–22%) in the presence of bacterial cells (Table 3). In less concentrated bacterial suspensions, the peptide tended to adopt a coiled-coil structure, but as the number of bacterial cells increased, the formation of an isolated α-helix was observed. With the increase in the bacterial cells/peptide ratio, the surface area of interaction of the lipopeptide with the cell membrane increases, which may affect the forces of interaction inside the coiled-coil structures and lead to the disintegration of peptide assemblies. 

### 2.5. Effect of Lipopeptides on the Phase Transition Temperature of DPPC and DPPG Lipids

**Figure 3:**
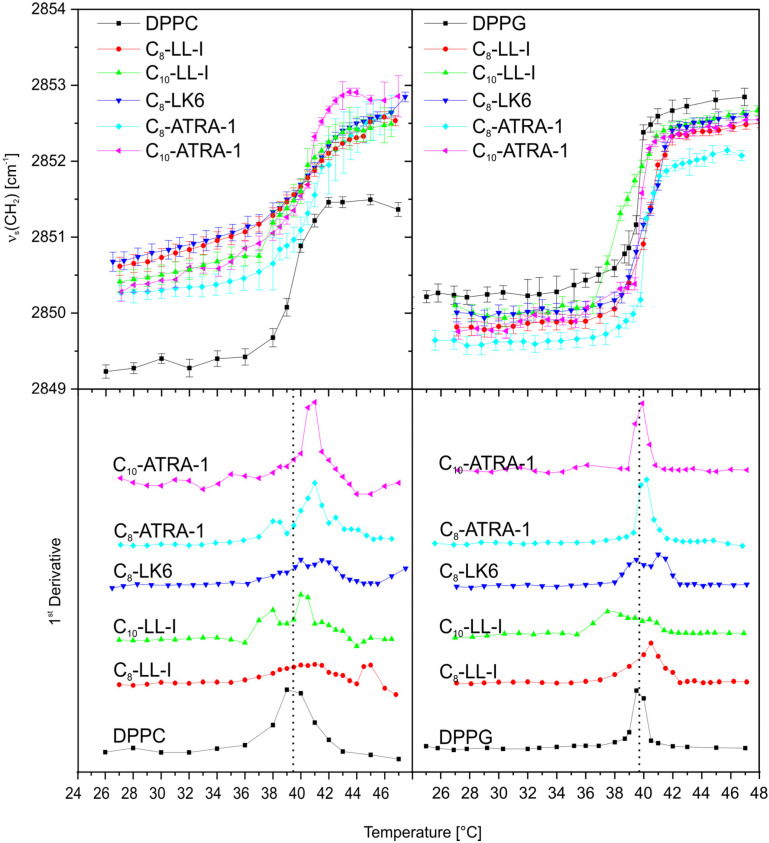
Evaluation of the maximum frequency of the v_s_(CH_2_) bands as a function of temperature (**top** panel) and 1st derivatives (**bottom** panel) of the relationships for DPPC, and DPPG in the absence and the presence of lipopeptides.

The changes in the structure of artificial membrane models, DPPC (phospholipids, 1,2-dipalmitoyl-*sn*-glycero-3-phosphocholine) and DPPG (1,2-dipalmitoyl-*sn*-glycero-3-[phospho-rac-(1-glycerol)]) induced by the lipopeptides were monitored on FTIR spectra by tracking the position of symmetric CH_2_ stretching vibrations of the lipid methylene groups, v_s_(CH_2_), as a function of temperature (Figure 3). In all cases the lipids underwent a phase transition from an ordered gel phase to a liquid crystalline phase, which was characterized by un upward shift of v_s_(CH_2_) bands by ca. 3 cm^−1^. The inflection point in the v_s_(CH_2_) vs. temperature corresponded to a main phase transition temperature, T_m_. In the case of neat DPPC and DPPG membrane models, T_m_ was found at ca. 39.5 °C. Premixing of the DPPC and DPPG vesicles with lipopeptides induced T_m_ changes ranging from −2 to 1.5 °C. However, with C_8_-LL-I in DPPC, a shoulder observed at 45 °C on the first derivative curve indicated an increase in the T_m_ even by 5.5 °C. The appearance of more than one maximum on the first derivative curve as well as the broadening of the phase transition suggest differential distribution of the peptides in the lipid bilayer.

With negatively charged DPPG, a rise in the T_m_ temperature is provoked by electrostatic interactions between peptide and lipid headgroups, which reduce the repulsion between adjacent lipids, leading to closer contact between their acyl chains and consequently to their higher ordering. In turn, an insertion of molecules into the membrane driven by hydrophobic interactions typically leads to a decrease in the main phase transition temperature [37]. In the case of the studied lipopeptides, the relatively minor changes in the T_m_ indicated more or less compensation for the electrostatic and hydrophobic interactions. This was particularly evident in the case of ATRA-1 derivatives, which despite the highest overall positive charge, did not induce changes in the T_m_ of the DPPG or induced changes that were negligible below the resolution of the experiment. In turn, when comparing C_8_-LL-I with C_10_-LL-I, it was observed that elongation of the hydrocarbon chain of the fatty acid shifted the T_m_ of DPPG to a lower value. This phenomenon confirmed an increase in the contribution of the hydrophobic interaction in the binding process. Additional information was deduced from the absolute values of the CH_2_ stretching vibrational frequencies. As can be seen in Figure 3, the lipopeptides initiated a shift of the CH_2_ stretching vibrations to lower wavenumbers over the entire temperature range compared to the neat DPPG. This phenomenon, together with an elevated T_m_, confirmed a higher order of the lipid acyl chains and/or enhanced interchain vibration coupling. In turn, with the DPPC complexes, the symmetric CH_2_ stretching bands were shifted to higher wavenumbers in both the gel and liquid-crystalline phases, which is usually related to higher membrane fluidity and/or reduced interchain vibrational coupling [38]. Both phenomena reflect disturbances in the lateral interactions between phospholipid chains in the complexes either due to increases in the number of *gauche* conformers in the hydrocarbon chains or their looser packing. In addition, the lipopeptides induced phase separation upon binding to the DPPC, leading to the formation of the lipid domains with different melting temperatures. The increase in the frequency of the CH_2_ stretching vibrations over the whole temperature range correlated well with the existence of the lipid domains with reduced T_m_. Both findings confirmed increased fluidity of the DPPC lipid bilayer. On the other hand, the appearance of the domains with the T_m_ above that of the neat DPPC implied a stabilization of gel-phase, which may be due to the dehydration of the membrane interface at the peptide binding site [39,40]. Moreover, above the T_m_, the choline groups of the DPPC are folded inward toward the surface, exposing more of the negatively charged phosphatidyl groups [41]. Thus, the possibility of electrostatic interactions between the positively charged lipopeptides and negatively charged phosphatidyl groups is rising.

### 2.6. The Thermodynamic Parameters of Lipopeptide Binding to the Lipid Bilayer

The results of the isothermal titration calorimetry (ITC) studies showed no, or more likely, too weak interactions between the lipopeptides and POPC, undetectable by the ITC method. The exception was C_10_-LL-I (Figure S4, Supplementary Material), which bound to the POPC vesicles with a negative enthalpy change (Δ*H* < 0) and a binding constant of 3.8·10^5^ M^−1^ (Table 4). A closer inspection of the experimental data confirmed a spontaneous (Δ*G* < 0) and entropy-driven (|Δ*H*| < |TΔ*S*|) process. Entropic control of the binding process indicated that hydrophobic interactions play a predominant role in the association of the peptide with zwitterionic POPC liposomes. In turn, all the ITC experiments with POPG liposomes confirmed the interactions of the lipopeptides with anionic lipids but with different thermodynamic effects. Thus, the titration of POPG to C_8_-LK6 and both ATRA-1 derivatives began with endothermic heat pulses accompanying the initial injections, becoming exothermic after several steps. These biphasic isotherms may suggest that the first stage of interaction was dominated by a release of a large number of water molecules both from the lipid bilayer and the peptide surfaces, which resulted in a large entropy gain. In turn, the exothermic effects following subsequent titration steps may be attributed to the peptide–membrane electrostatic attraction and peptide folding. On the other hand, the peptide–membrane association can occur in parallel with other processes, such as a change in the lipid phase properties, increase in permeability of the membrane, micellization of the lipid bilayer or initial peptide aggregation [42]. Despite the biphasic isotherms, better fitting results were achieved using the “one set of site” binding model, which showed that the overall process was dominated by an unfavorable enthalpy change (Δ*H* > 0) offset by a large, favorable entropy change (TΔ*S* > 0). With C_8_-LK6, the ITC experiment repeated with a twice higher initial peptide concentration (Figure S4, Supplementary Material) highlighted even more complexity of the peptide–membrane interactions. However, computing accurate thermodynamic parameters for multiple binding events of many different affinities was not possible. Different results were obtained for the LL-I analogues. Namely, the titration of POPG into the peptide solutions resulted in a heat release over the whole concentration range. Unexpectedly, the binding of C_8_-LL-I to POPG vesicles was enthalpy-driven (|Δ*H*| > |TΔ*S*|), which indicated a significant role of electrostatic and van der Waals interactions and hydrogen bonds in the membrane association. In turn, an unfavorable entropic contribution to the free energy of binding (TΔ*S* = −3.10 ± 1.36 kcal/mol) may suggest an enhanced ordering of the peptide–POPG complex. Titration of the POPG into the C_10_-LL-I solution involved a two-step binding process. An analysis of the isotherm with the “two sets of sites” model confirmed that both processes were spontaneous (Δ*G* < 0) and entropy-driven (|Δ*H*| < |TΔ*S*|), but the determined parameters were burdened with a large error. 

### 2.7. Transmission Electron Microscopy Imaging

**Figure 4:**
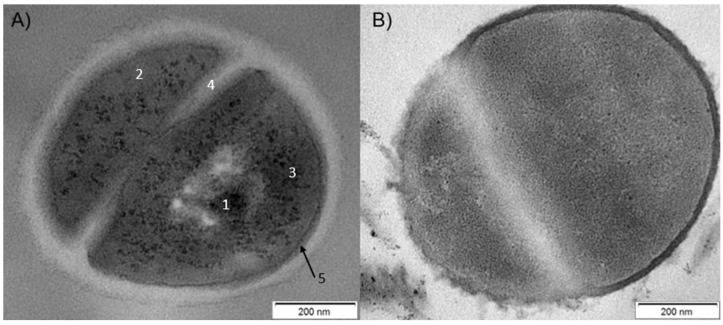
Transmission electron microscopy (TEM) images of *S. aureus* cells; (**A**) negative control—untreated dividing cells; 1—nucleoid, 2—cytoplasm, 3—DNA strands, 4—septum, 5—cell membrane; (**B**) cells treated with C_8_-LL-I—visible disintegration of cell membrane is observed.

Transmission electron microscopy was employed to visualize the effect of the representative analogue, C_8_-LL-I, on the *S. aureus* cells (Figure 4). The TEM micrograph of the untreated *S. aureus* cell showed a typical morphology with a relatively thick and, without doubt, intact cell wall. In turn, incubation of the *S. aureus* cells with C_8_-LL-I induced thinning of the membrane and its disintegration, as evidenced by its irregular shape. The results unambiguously confirmed that the destruction of the bacterial membrane is the primary effect that underlies the biological activity of the tested lipopeptides.

### 2.8. Self-Assembly via Molecular Dynamics Simulations

The α-helical peptides are promising self-assembling building blocks in a bottom-up nanomaterial design strategy. Inspiration comes from inter-helical interactions that play an important role in the proper folding of many proteins, especially those attached to or associated with membranes [43,44,45,46]. Going further, conjugation of the α-helical peptide with a fatty acid increases the hydrophobicity of the compound, enhancing a tendency to self-assemble in solution and in membranes. Therefore, to take a deeper look at the self-assembly at the molecular level, full-atom self-assembly simulations were performed for the representative lipopeptides, C_8_-LL-1, C_8_-LK6 and C_10_-ATRA-1 (Figures S5–S7, Supplementary Material). 

**Figure 5:**
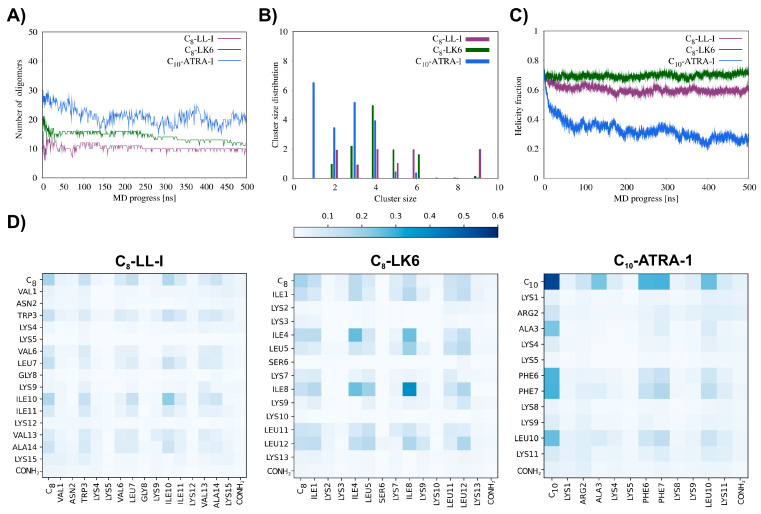
Analysis of the 500 ns MD simulations following the aggregation of the three representative lipopeptides, C_8_-LL-I, C_8_-LK6 and C_10_-ATRA-1. (**A**) The number of oligomers over the MD simulation time. (**B**) The cluster size distributions over the last 50 ns of the MD simulations. (**C**) The overall helicity over the MD simulation time. (**D**) The inter-residue contact map with probabilities according to the color scale. Contacts were determined between all the peptides in the 50-molecule system in the last 50 ns of the 500 ns MD simulations.

The lipopeptides started to self-assemble in the first steps of the simulations to form oligomers with a number of aggregations up to nine (Figure 5). The number of oligomers stabilized after approximately 250 ns of MD simulations for C_8_-LL-I and C_8_-LK6. With C_10_-ATRA-1, large fluctuations in the number of oligomers were observed until the end of the 500 ns MD simulations. 

**Figure 6:**
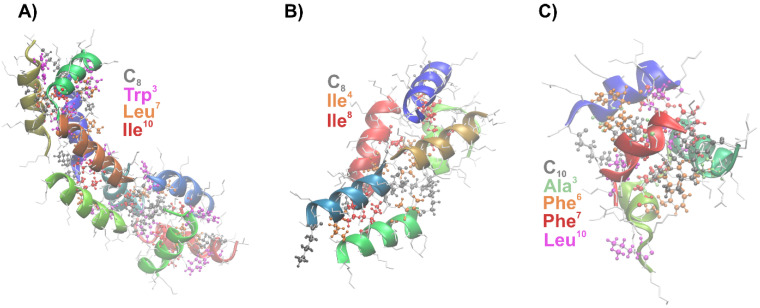
The largest oligomers extracted from the final snapshots of the 500 ns full-atom self-assembly simulations of (**A**) C_8_-LL-I, (**B**) C_8_-LK6 and (**C**) C_10_-ATRA-I. The backbone is represented as a ribbon model. The residues most often involved in hydrophobic interactions are presented in the ball-and-stick model.

Both C_8_-LL-I and C_8_-LK6 self-assembled into a fibrillar morphology, producing staggered packing of α-helices, which allowed for axial growth of coiled-coil oligomers (Figure 6). The interstrand hydrophobic interactions included most of all the N-terminal fatty acid chain and amino acid side chains in the central region of the peptides (Figure 5D). These interactions stabilized the α-helical structure of the C_8_-LL-I and C_8_-LK6 during the MD simulations. In turn, C_10_-ATRA-1, despite the presence of the Phe-Phe motif [47] in the sequence, which is considered sufficient to promote self-assembly, showed the lowest self-assembly tendency among the tested lipopeptides. On the other hand, the ATRA-1 derivative had the highest overall positive charge among the tested peptides, which is not conducive to self-assembly. Therefore, for C_10_-ATRA-1, a large fraction of molecules in the monomeric state was reported after the 500 ns MD simulations, which promoted peptide unfolding and about a two-fold decrease in the overall helical fraction compared to the initial configuration. Among the oligomers, the most popular were those composed of 2–4 units. The trimers and tetramers adopted a globular shape rather than a fibrous structure with major hydrophobic interactions occurring between the decanoyl chains of adjacent molecules (Figure 5D). 

### 2.9. Peptide-Membrane via Coarse-Grained Molecular Dynamics Simulations

**Figure 7:**
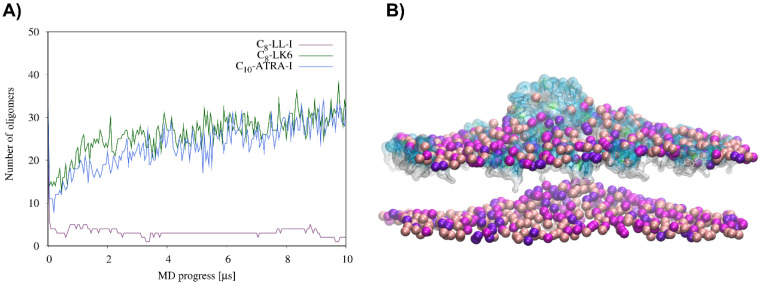
(**A**) Analysis of the self-assembly process of the lipopeptides in the presence of the DPPG/LysPG (lysyl-phosphatidylglycerol)/CDL2 (cardiolipin) bilayer as a function of simulation time. Two peptide molecules were classified into the same oligomer if any of their beads fell within the distance of 6 Å of each other. (**B**) Snapshot from the DPPG/LysPG/CDL2 binding CG MD simulations for C_8_-LK6, showing the lipopeptide micelle insertion at 9 µs. For clarity, only phosphate beads of the membrane are displayed. The DPPG, LysPG and CDL2 phosphate groups are indicated in pink, magenta and violet, respectively. Oktanoyl tails and nonpolar amino acids are in white, positively charged residues are in cyan, while polar noncharged residues are in green.

Coarse-grained molecular dynamics simulations were used to investigate the interactions of the representative lipopeptides, C_8_-LL-1, C_8_-LK6 and C_10_-ATRA-1, with the *S. aureus* bilayer model. To validate the CG MD simulations, the results were compared to the intact lipid bilayer (Figures S8–S11, Supplementary Material). In the first steps of the CG MD simulations, the lipopeptide monomers were attracted to the lipid bilayer or self-assembled into oligomers and/or micelles, which finally approached the membrane surface anyway. A detailed analysis of the results showed an expected drop in the number of clusters at the beginning of the CG MD simulations due to the lipopeptide self-assembly (Figure 7A). However, for the C_8_-LK6 and C_10_-ATRA-1 systems, the number of clusters started to increase again, first relatively quickly (up to 600 ns) and then slowly, reflecting their insertion and gradual dispersion of the monomers in the outer leaflet of the membrane. Finally, all the C_8_-LK6 molecules and about 80% of the C_10_-ATRA-1 molecules penetrated into the lipid bilayer. Incorporation of the peptide’s micelle into the lipid bilayer began with its gradual opening from the membrane-bound site, which was accompanied by a distinct bulge in both leaflets of the membrane (Figure 7B). This phenomenon excluded polar residues from the contact region and reduced the electrostatic energy barrier for mixing hydrophobic tails of lipopeptides and membrane lipids. After complete insertion, the peptide molecules rapidly dispersed throughout the membrane, and the membrane itself flattened out. Finally, only negligible curvature was observed. By contrast, the C_8_-LL-I worm-like micelle, created quickly at the beginning, remained bound to the membrane surface until the end of the 10 µs CG MD simulations.

**Figure 8:**
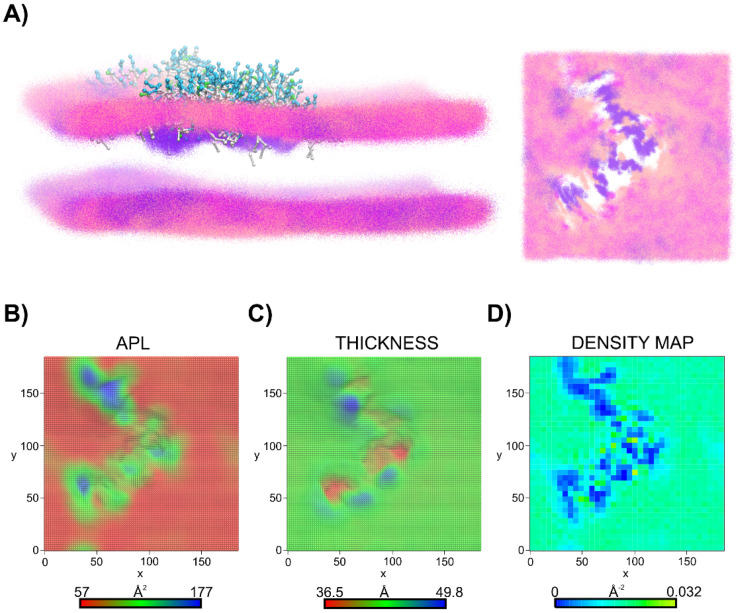
C_8_-LL-I induced membrane thinning in the DPPG/LysPG/CDL2 bilayer. (**A**) Superimposed snapshots from the last 500 ns of the DPPG/LysPG/CDL2 binding CG MD simulations for C_8_-LL-I. For clarity, only the phosphate beads along the trajectory part from 9.5 to 10 µs are shown, while the worm-like lipopeptide micelle was only taken from the final snapshot (**left** view). To better visualize the holes caused by C_8_-LL-I in the upper leaflet of the membrane, the lipopeptide molecules were removed (**right** view). The DPPG, LysPG and CDL2 phosphate groups are indicated in pink, magenta and violet, respectively. Oktanoyl tails and nonpolar amino acids are in white, positively charged residues are in cyan, while polar noncharged residues are in green. (**B**) Area per lipid (APL) map in the upper leaflet of the DPPG/LysPG/CDL2 membrane (top view), and (**C**) membrane thickness map (top view). (**D**) Two-dimensional density map of all the lipids in the upper leaflet of the DPPG/LysPG/CDL2 membrane (top view).

Surface-bound micelles induced an evident segregation of membrane lipids, leading to a distinct enrichment of the micelle binding site in cardiolipin (CDL2) (Figures S9–S11, Supplementary Material). Following this, the increase in CDL2 density promoted the negative membrane curvature, a phenomenon well seen in the system with C_8_-LL-I (Figure 8A). This also translated into changes of area per lipid (APL) and thickness of the membrane at the binding site (Figure 8B,C and Figure S9C Supplementary Material). A deep analysis even showed a 2.4-fold increase in APLs compared to the neat membrane and various changes in the membrane thickness (Figures S8 and S9, Supplementary Material). The center of the membrane space directly beneath the micelle was characterized by reduced thickness, and its periphery by increased thickness, with a difference of up to 13 Å. The perturbated bilayer structure was also evident from the two-dimensional lipid density map in the upper leaflet of the membrane. As can be seen, the peptide disrupted the membrane integrity (Figure 8). 

Regardless of whether the positively charged lipopeptides were fully inserted into the membrane or only bound to it in an aggregated form, they affected the electrostatic state of the outer membrane–water interface. Moreover, they contributed to the displacement of sodium ions and changes in their concentration in the extracellular space. All this should lead to disruption of the electrostatic potential across the membrane (Figures S9–S11, Supplementary Material), and consequently to the disturbance of the behavior and function of bacterial cells.

## 3. Materials and Methods 

### 3.1. Reagents

All amino acid residues and coupling reagents were purchased from GL Biochem (Shanghai, China). The fatty acids: caprylic acid, capric acid, lauric acid, myristic acid and palmitic acid, were acquired from Merck (Darmstadt, Germany), Sigma-Aldrich (Darmstadt, Germany) and Acros Organics (Geel, Belgium). The resin and solvents were purchased from Rapp Polymere (Tübingen, Germany) and Merck (Darmstadt, Germany). The phospholipids, 1,2-dipalmitoyl-sn-glycero-3-phosphocholine (DPPC), 1,2-dipalmitoyl-sn-glycero-3-[phospho-rac-(1-glycerol)] (DPPG), 1-palmitoyl-2-oleoyl-glycero-3-phosphocholine (POPC) and 1-palmitoyl-2-oleoyl-sn-glycero-3-[phospho-rac-(1-glycerol)] (POPG) were purchased from Avanti Polar Lipids (Alabaster, AL, USA). The Mueller Hinton broth was bought from BIOCORP (Warsaw, Poland) and the RPMI-1640 medium from Sigma-Aldrich (Darmstadt, Germany). Bacterial strains *Staphylococcus aureus* PCM 1650, *Staphylococcus aureus* PCM 2054, *Staphylococcus epidermidis* PCM 521, *Staphylococcus epidermidis* PCM 2118, *Escherichia coli* PCM 2057 obtained from the Polish Collection of Microorganisms (PCM, II TD PAN, Wrocław), *Candida albicans* PCM 2566, *Candida glabrata* CCM 8270, *Candida krusei* CCM 8271, *Candida parapsilosis* CCM 8260 were purchased from Czech Collection of Microorganisms (CCM, Department of Experimental Biology Faculty of Science, Masaryk University, Brno).

### 3.2. Peptide Synthesis

**Scheme 1:**
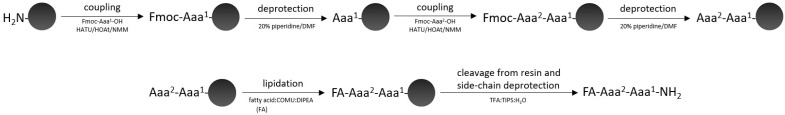
Solid-phase peptide synthesis.

All the peptides were synthesized on a Tenta Gel S RAM resin (0.22 mmol/g) according to previously described methodology [42]. After deprotection of the N-terminal part of the peptide chain with 20% piperidine in N,N-dimethylformamide (DMF), the modifications were performed using fatty acids with various carbon chain lengths (C_8_–C_16_), (1-cyano-2-ethoxy-2-oxoethylidenaminooxy)dimethylamino-morpholino-carbenium hexafluorophosphate (COMU) and N,N-diisopropylethylamine (DIPEA) of molar ratio peptide:fatty acid:COMU 1:3:3 (pH 8–9). Coupling of fatty acid was carried out for 2 × 15 min. Cleavage of the final peptides from the resins was performed using a mixture of trifluoroacetic acid (TFA), triisopropylsilane (TIPS) and water (95:2.5:2.5 *v*/*v*/*v*) for 2 h at room temperature (Scheme 1). Then, the resins were drained, and the obtained solutions were concentrated. The crude peptides were precipitated by the addition of diethyl ether and centrifuged; the films were then dried in a stream of nitrogen gas, dissolved in water and lyophilized.

The synthesized analogues were purified by the Waters RP-HPLC preparative system with Jupiter 4 μm Proteo column, 90 Å, 250 × 10 mm (Phenomenex, Torrance, CA, USA). The linear gradient (B in A) from 10 to 100% within 60 min (A: 0.1% solution of TFA in water, B: 80% solution of acetonitrile in A) and with a flow rate of 5 mL/min. The purity of the lipopeptides (at least 95%) was performed by the PR-HPLC analytic system with Jupiter 4 μm Proteo column, 90 Å, 250 × 4.60 mm (Phenomenex, Torrance, CA, USA). The linear gradients were from 1 to 80% B, 15 to 90% B and 50 to 100% B within 30 min and with a flow rate of 1 mL/min.

The identity of final products was carried out by recording the molecular mass by mass spectrometry (MALDI-TOF, Biflex III, Bruker, Karlsruhe, Germany). Analytical data of all the lipopeptides are presented in Table S2, Supplementary Materials. 

### 3.3. Antimicrobial Activity Assays

The evaluation of the antimicrobial activity of the synthesized lipopeptides was performed against the following reference bacterial and fungal strains: *Staphylococcus aureus* PCM 1650, *Staphylococcus aureus* PCM 2054, *Staphylococcus epidermidis* PCM 521, *Staphylococcus epidermidis* PCM 2118, *Escherichia coli* PCM 2057, *Candida albicans* PCM 2566, *Candida glabrata* CCM 8270, *Candida krusei* CCM 8271, *Candida parapsilosis* CCM 8260.

For the initial cultivation of the bacteria and fungi, Luria-Bertani broth (LB, Biomaxima, Lublin, Poland) and Sabouraud Dextrose broth (SB, Biomaxima, Lublin, Poland) were used, respectively. Antimicrobial properties of the compounds were analyzed through the determination of MIC (minimal inhibitory concentrations) by my means of the serial micro-dilution technique according to the guidelines of Clinical & Laboratory Standard Institute (CLSI) established for aerobic bacteria [48] and yeasts [49]. 

According to the procedures, Mueller–Hinton broth (MHB) was used as the working medium for the analysis of the bacterial susceptibility, while RPMI-1640 (with L-glutamine, without sodium bicarbonate) growth medium buffered with MOPS (morpholinepropanesulfonic acid) and supplemented with 2% glucose was used for the experiments with the fungi. 

The minimum inhibitory concentration values (MIC) were determined by exposing the bacteria or fungi cultures with an initial inoculum of 5 × 10^5^ and 5 × 10^3^ CFU/mL, respectively, to the increasing concentrations of the serially diluted tested lipopeptide applied in the range of 6.25–50 µg/mL. The experiments were carried out for 24 h at 37 and 35 °C for the bacteria and fungi, respectively. Then, the absorbance was measured at 600 nm for each well of the 96-well plate using a microplate reader (Sunrise TM, Tecan Trading AG, Männedorf, Switzerland). Based on the obtained results, the degree of inhibition of the growth of microorganisms (expressed in %) in the presence of the tested compounds range was determined with respect to the control probes. Inoculated wells without peptide were treated as a positive control (100% growth), and the wells containing working medium only were defined as a negative control (0% growth). All measurements were repeated in triplicate.

### 3.4. MTT Assays

MTT assay was used to determine the cytotoxicity of the studied compounds toward breast cancer cells (MCF-7 line, ATCC), prostate cancer cells (PC3 line, ATCC) and human keratinocytes (HaCaT line, ATCC). The MCF-7 cell line was cultured in a RPMI 1640 medium (Gibco, ThermoFisher Scientific, Waltham, MA, USA), the PC3 line in F-12K medium (Gibco, ThermoFisher Scientific, Waltham, MA, USA) and the HaCaT line in the high-glucose DMEM medium (Gibco, ThermoFisher Scientific Waltham, MA, USA). All media were supplemented with 10% fetal bovine serum (Gibco, ThermoFisher Scientific, Waltham, MA, USA) and antibiotics (penicillin and streptomycin, Gibco, ThermoFisher Scientific, Waltham, MA, USA). Cells were seeded in 96-well plates at a density of 4000 per well and incubated at 37 °C in a 5% CO_2_ atmosphere. Then, the cells were treated with tested derivatives in the concentration range of 0.1 to 200 µg/mL (or 0.1–100 µg/mL in the case of C_8_-LK6 due to its limited solubility) or with the same volume of the vehicle (H_2_O) alone as a control. The prepared plates were further incubated for 48 h. After this time, the aqueous MTT salt solution (3-(4,5-Dimethylthiazol-2-yl)-2,5-diphenyltetrazolium bromide, VWR) in a concentration of 4 mg/mL was added (25 µL/well). After 3 h of incubation, the formazan product was dissolved in DMSO, and the absorbance was measured at 570 nm (the background absorbance was measured at 660 nm) using an EnSpire microplate reader (Perkin Elemer, Waltham, MA, USA). The control cell viability was assumed to be 100%. Three independent experiments were carried out in three repetitions. The statistical analysis of results and IC_50_ values estimation were performed using GraphPad Prism 7 software (San Diego, CA, USA).

### 3.5. Liposomes Preparation

Liposomes made of POPG (1-palmitoyl-2-oleoyl-*sn*-glycero-3-[phospho-rac-(1-glycerol)] and POPC (1-palmitoyl-2-oleoyl-*sn*-glycero-3-phosphocholine) were obtained by hydration of the dry lipid film. For this purpose, the lipids were dissolved in a mixture of chloroform and methanol in a volume ratio of 4:1. The solvents were evaporated in the stream of nitrogen gas, and then, the samples were lyophilized to remove residual organic solvents. Afterward, the lipid film was hydrated with phosphate-buffered saline (PBS, pH 7.4) and shaken for 2 h at 60 °C. Subsequently, the obtained multilamellar vesicles (MLVs) suspension was subjected to several cycles of freezing in liquid nitrogen and thawing at 60 °C to reduce the size and layers of the liposomes. Large unilamellar vesicles (LUV) were obtained by extrusion of MLV liposomes through polycarbonate membrane filters with a pore diameter of 100 nm (Whatman International Ltd., Kent, UK) using a mini-extruder (Avanti Polar Lipids, Inc., Alabaster, AL, USA). 

In the FTIR measurements, MLVs built with DPPC (1,2-dipalmitoyl-*sn*-glycero-3-phosphocholine) and DPPG (1,2-dipalmitoyl-*sn*-glycero-3-[phospho-rac-(1-glycerol)]) were prepared by dissolving the lipids in a mixture of chloroform and methanol in a volume ratio of 2:1, dried and lyophilized. The obtained films were resuspended in a phosphate buffer (pH 7.4) and mixed for 2 h at 60 °C. After this, the samples were frozen and thawed 5 times. The prepared MLVs were blended with the lyophilized lipopeptides to obtain the desired peptide-to-lipid 1:10 molar ratio and incubated for 1 h at room temperature.

### 3.6. Circular Dichroism (CD)

CD experiments were carried out at 25 °C using a Jasco J-815 spectropolarimeter in the wavelength range 185–260 nm. The concentration of the lipopeptides was 0.15 mg/mL. The CD spectra were recorded in water, PBS (pH 7.4), 1.3 mM POPC and 1.3 mM POPG. The results are plotted as the mean molar ellipticity per one amino acid residue (MRME, deg cm^2^ dmol^−1^) as a function of the wavelength (nm). Additionally, we performed a conformational analysis of the selected compounds in the presence of *S. aureus* cells. In this case, 0.15 mg/mL solutions of the tested peptides were prepared in the phosphate buffer (10 mM, pH 7.0) and inoculated with bacteria (5 × 10^6^ CFU/mL), cultivated the same way as for the MIC experiment. The mixtures were then incubated for 2 h before recording the CD spectra. 

The percentage of the α-helical structure was calculated based on the mean molar ellipticity at 222 nm using the formula:

$$f_{H}=\frac{\theta_{222}-3000}{-36000-3000}\cdot 100\%$$

where θ_222_ corresponds to the value of the mean molar ellipticity at a wavelength of 222 nm [50]. 

### 3.7. Isothermal Titration Calorimetry (ITC)

The thermodynamic parameters of the interactions of the lipopeptides with POPG and POPC liposomes were determined by isothermal titration calorimetry (ITC). Measurements were performed at 25 °C using an AutoITC microcalorimeter (MicroCal Inc., Northampton, MA, USA) with a 1.4491 mL sample and reference cells. The experiment consisted in the titration of a buffered peptide solution with a concentration of 0.05 and/or 0.07 mM with a buffered solution containing POPG or POPC LUVs with a lipid concentration of 1.3 and 1.8 mM (10.02 µL; 29 injections, 2 µL for the first injection only), as it was described previously [51]. The result of the titration was a thermogram showing a series of peaks corresponding to the titration steps. A titration curve was obtained after integration of the peak areas. The obtained isotherms were fitted to the “one set of sites” or “two sets of sites” models available in the ORIGIN 7.0 (MicroCal Inc., Northampton, MA, USA), which made it possible to determine the enthalpy (Δ*H*), the binding constant (K_ITC_) and the stoichiometry (N) of the interactions between the lipopeptide and the lipids. The following formula was used to calculate the entropy change (Δ*S*) and the Gibbs free energy (Δ*G*):

$$\Delta G=-\mathrm{RTln}\left(55.5K_{\mathrm{ITC}}\right)=\Delta H-T\Delta S$$

where: factor 55.5 is the molar concentration of water, R is the gas constant (1.986 cal·mol^−1^·K^−1^), and T is the absolute temperature. 

### 3.8. FTIR Measurements

Fourier transform infrared spectra were recorded on a IFS66 spectrometer (Bruker, Billerica, MA, USA) equipped with a DTGS detector. The samples were placed between two CaF_2_ windows separated with a 50 µm Teflon spacer. The spectra were measured between 3000 and 2500 cm^−1^ at 2 cm^−1^ resolution as the average of 10 measurements each with 16 scans. The cuvette was thermostated to the appropriate temperature in the range of 27–46 °C by using a Julabo F33 thermostat (Seelbach, Germany), and the sample temperature was controlled with a CHY502 thermometer. The spectrum of the phosphate buffer solution at the appropriate temperature was each time subtracted from the final spectra. The wavenumber positions for symmetric and asymmetric CH_2_ stretching modes of the lipid hydrocarbon chains were measured using a multiple Gaussian curve fitting procedure in OriginPro 2021 software. Finally, the frequency of the symmetrical CH_2_ stretching modes was plotted against the temperature. For a more precise determination of the temperature of the main phase transition of the lipids, T_m_, the first derivatives of the relationships were calculated. The main phase transition temperature of the DPPC and DPPG was determined before and after binding of the lipopeptides. 

### 3.9. Dynamic Light Scattering (DLS)

The hydrodynamic diameters of the lipopeptide nanoaggregates were examined by the Litesizer 500 (Anton-Paar GmbH, Graz, Austria) at 25 °C. The measurements were carried out at various lipopeptide concentrations ranging from 0.5 to 2 mM and from 0.25 to 2 mM, in water and PBS, respectively. Two-fold serial dilutions were used to obtain the sequential concentrations. Each sample was sonicated for 10 min followed by incubation for 1 h at 25 °C and transferred to the polystyrene cuvette. The detector was positioned at a side scatter position (90°). The analysis of the obtained results and report generation was performed with KalliopeTM software (Anton-Paar GmbH, Graz, Austria).

### 3.10. Transmission Electron Microscopy (TEM)

TEM was used to observe ultrastructural changes in *S. aureus* cells in the presence of C_8_-LL-I. Bacterial cells were incubated overnight in a Mueller–Hinton medium. The cells were centrifuged (8000 rpm, 15 min) and resuspended in fresh media to obtain a high-density cell suspension (5 × 10^5^ CFU/mL). C_8_-LL-I in a MIC concentration (25 µg/mL) were mixed with the cells and incubated overnight at 37 °C. Afterward, the cells were centrifuged (8000 rpm, 15 min) and washed three times with PBS. The bacterial cells were fixed with 2.5% glutaraldehyde buffered at pH 6.5 with 0.1 M sodium cacodylate (Polysciences, Warrington, PA, USA) for 6 h at room temperature, as described earlier [52]. Additional fixation was performed with a 1% osmium tetroxide solution (Polysciences, Warrington, PA, USA) for 2 h at 4 °C. The cells were centrifuged and resuspended in 0.1 M cacodylate buffer, centrifuged again and dehydrated with ethanol. The bacteria were embedded in Epon 812 resin (Sigma-Aldrich, Darmstadt, Germany) at room temperature. A Leica UC7 ultramicrotome (Wetzlar, Germany) was used to obtain ultra-thin sections (55 nm). As contrasting agents, lead citrate and uranyl acetate were used. Cells were visualized using a Tecnai Spirit BioTWIN (EFI, Hillsboro, OR, USA) microscope at 120 kV.

To obtain TEM micrographs of the formed nanostructures, peptide samples in the concentration of 2 mg/mL were incubated in PBS at 37 °C for 24 h. Then, 5 µL of an appropriate sample was applied on carbon-coated copper grids (300 mesh), and after absorption time, the sample was stained with 2% (*v*/*v*) aqueous uranyl acetate. The micrographs were obtained using the same device and conditions as described above.

### 3.11. The Self-Assembly Simulations

The all-atom molecular dynamic simulations were used to trace the self-assembly properties of the selected lipopeptides. The simulations were performed using the GPU/CUDA-accelerated implementation of PMEMD in AMBER 18 [53]. Non-standard fatty acid residues were modeled with XLEAP. The point charges were optimized by fitting them to the ab initio molecular electrostatic potential (6–31G* basis set, GAMESS 2013—ab initio molecular electronic structure program) [54] for two different conformations, followed by consecutive averaging of the charges over all conformations, as recommended by the RESP protocol [55]. The initial configuration was constructed by randomly placing 50 peptide molecules in a simulation box using PACKMOL [56]. The system was solvated and neutralized by adding chloride ions using the LEAP program available in the AMBER 18 package and minimized for 40,000 steps (steepest descent method) with weak harmonic constraints on the peptide molecules (10 kcal/mol Å) used for the first 10,000 steps. Afterward, the system was equilibrated using a three-step protocol: (1) 10 ns simulations with isotropic pressure coupling and a 0.5 fs time step, (2) 10 ns simulation with isotropic pressure coupling and a 1 fs time step, and (3) 500 ns simulations with isotropic pressure coupling and a 2 fs time step. Periodic boundary conditions were employed in all the simulations. Long-range electrostatic interactions were evaluated by the particle Mesh Ewald (PME) summation. A cut-off of 10 Å was used for van der Waals interactions. The SHAKE algorithm was used to constrain bonds involving hydrogen. The temperature was maintained using the Langevin coupling scheme with a friction coefficient of 1 ps^−1^, whereas a Berendsen barostat maintained a reference pressure set to 1.0 bar. The analysis of the MD trajectories was performed by AMBER 18 [53] and GROMACS 2019.5 tools [57]. The inter-residue contact maps were created with Python-based scripts available as a public repository on GitHub (https://github.com/strodel-group/Oligomerization-State_and_Contact-Map, accessed on 14 November 2022). Visualizations were performed with VMD 1.9.4a38 [58].

### 3.12. CG MD Simulations of Spontaneous Peptide-Membrane Interactions

Molecular dynamics simulations were carried out using the GROMACS 2019.5 package [57] and the MARTINI coarse-grained force field [59,60]. The lipid bilayer was composed of 54% DPPG, 36% Lys-PG (lysyl-phosphatidylglycerol) and 5% CDL2 (cardiolipin) equally distributed between two membrane leaflets to mimic the membrane of *S. aureus* [61]. The system was built using the insane.py script available on the martini website (http://cgmartini.nl/, accessed on 1 July 2022). The system was solvated and neutralized by adding counterions using the GROMACS tools. The salt concentration for bulk solution was 100 mM NaCl. The system was energy-minimized and equilibrated with the stepwise lowered force constant of the harmonic restraints (from 200 to 10 kJ mol^−1^ nm^−2^) to fix the position of the headgroups of the membrane lipids during simulations. After equilibration, the system was subjected to isothermal–isobaric molecular dynamics (NTP) with a 10 fs time step, as suggested by Wigner et al. [62] The temperature was held at 323 K using the v-rescale temperature coupling. The pressure was treated semiisotropically at 1 bar using the Parinello–Rahman barostat with a coupling constant of τ_p_ = 12.0 ps. The relative dielectric constant for explicit screening was 15. Coulomb interactions were treated using a reaction field and a cutoff of 11 Å. Periodic boundary conditions were applied in all directions. In the next step, the *S. aureus* lipid bilayer after 5 µs of CG MD simulations was used to build the systems with the C_8_-LL-I, C_8_-LK6 and C_10_-ATRA-1 lipopeptides. The lipopeptide molecules were placed randomly close to the outer leaflet of the membrane and neutralized by adding chloride ions. Details of the initial systems construction are given in Table S3 (Supplementary Materials). The peptide–membrane systems were minimized and equilibrated with the harmonic restraints (10 kJ mol^−1^ nm^−2^) to fix the position of the headgroups of the membrane lipids and peptides during the initial simulations. Afterward, the system was subjected to 10 µs CD MD simulations with the conditions mentioned above. However, to prevent peptide molecules drifting away from the outer membrane–water interface and binding to the inner leaflet due to the periodic boundary conditions, flat bottom harmonic restraints were applied between each peptide molecule and membrane. 

The data analysis was performed with *g_lomepro* software [63] and standard tools of the GROMACS. Visualizations were created in VMD 1.9.4a38 [58]. 

## 4. Conclusions

The α-helical lipopeptides presented in this study were characterized by high antimicrobial activity against a diverse range of Gram-positive bacteria and fungi from *Candida* spps. Based on the determined MIC values, C_8_-LL-I, C_10_-LL-I, C_8_-LK6, C_8_-ATRA-1 and C_10_-ATRA-1 were selected as the most promising compounds, and the *S. aureus* and *S. epidermidis* bacterial cells were selected as their main target. The cytotoxicity assays showed that the C_8_-ATRA-1 and C_10_-ATRA-1 analogues had relatively low cytotoxicity against human healthy keratinocytes but high cytotoxicity against the MCF-7 cell line. This could make them useful in the design of novel drug candidates active against breast cancer.

The tested lipopeptides demonstrated a strong tendency to self-assembly into fibrils and/or elongated and spherical micelles, while the size of the aggregates correlated well with cytotoxicity to the healthy keratinocytes. Among the lipopeptides with the highest antimicrobial potency, the C_10_-ATRA-1 analogue self-assembled into the smallest aggregates and at the same time revealed the lowest cytotoxicity. The ATRA-1 analogue carries the highest positive charge, suggesting that this feature affects self-assembly and contributes to cell selectivity. The C_10_-ATRA-1 analogue were also found to adopt a coiled-coil structure in the presence of the artificial lipid membranes. It is believed that the peptides folded in this way can be characterized by lower cytotoxicity [64]. 

The results of our study also confirmed that the bacterial cell membrane is the target for the studied compounds. The lipopeptides were able to penetrate the bacterial membrane and affect its properties, and monomers seemed to be the main active species. However, in silico studies showed that even the peptide assemblies that remained on the membrane surface induced drastic changes in the membrane permeability.

In summary, the main goal of our research was to design a novel group of peptides that form supramolecular assemblies and have biological activity. We chose α-helical antimicrobial peptides as the initial structures that were further derivatized with various types of fatty acids. The combination of nature-mimicking approaches inspired by the coiled-coil motif and the conjugation of helical peptides with hydrophobic alkyl chains to obtain peptide amphiphiles generated a wide repertoire of supramolecular hierarchic self-assembling architectures. Due to the combination of the physicochemical, in vitro and in silico analyses, our study contributes to the understanding of the role of the self-assembly in biological activity of the AMPs, which is of key importance for the potential use of this class of compounds in medicine and the pharmaceutical industry. Moreover, in our opinion, the obtained results expand the basic knowledge about peptide self-assembly, which is still poorly explored.

## Figures and Tables

**Table 1:** MIC (µg/mL) values determined for peptide derivatives.

Compounds	Gram(+)				Gram(-)	Fungi			
	*S. aureus* PCM1650	*S. aureus* PCM2054	*S. epidermidis* PCM521	*S. epidermidis* PCM2118	*E. coli* PCM2057	*C. albicans* PCM2566	*C. glabrata* CCM8270	*C. krusei* CCM8271	*C. parapsilosis* CCM 8260
**LL-I**	25	>50	12.5	6.25	12.5	>50	>50	50	50
**C_8_-LL-I**	12.5	12.5	6.25	6.25	25		25	12.5	12.5
**C_10_-LL-I**	12.5	12.5	12.5	6.25	25		25	12.5	6.25
**C_12_-LL-I**	12.5	12.5	12.5	6.25	>50		12.5	12.5	3.13
**C_14_-LL-I**	>50	>50	12.5	12.5	>50		12.5	12.5	3.13
**C_16_-LL-I**	>50	>50	25	25	>50		>50	>50	12.5
**LK6**	50	>50	6.25	6.25	>50	50	31.25	12.5	12.5
**C_8_-LK6**	12.5	12.5	6.25	6.25	>50	12.5	25	6.25	6.25
**C_10_-LK6**	>50	50	6.25	6.25	>50	6.25	6.25	6.25	6.25
**C_12_-LK6**	25	50	25	6.25	>50	6.25	3.13	6.25	6.25
**C_14_-LK6**	50	50	25	25	>50	6.25	12.5	6.25	6.25
**C_16_-LK6**	>50	>50	50	50	>50	6.25	>50	25	50
**ATRA-1**	>50	>50	25	50	>50	>50	>50	50	50
**C_8_-ATRA-1**	6.25	7.8	3.13	3.13	>50	>50	>50	12.5	50
**C_10_-ATRA-1**	6.25	3.9	3.13	3.13	31.25	50	>50	12.5	25
**C_12_-ATRA-1**	6.25	3.9	3.13	3.13	31.25	2.5	6.25	2.5	12.5
**C_14_-ATRA-1**	12.5	3.9	25	6.25	>50	2.5	3.13	2.5	3.13
**C_16_-ATRA-1**	>50	>50	25	12.5	>50	2.5	3.13	2.5	3.13

**Table 2:** IC_50_ values and selectivity indexes (SI) of studied compounds.

Compounds	IC_50_ (µg/mL)			SI ^1^	
	MCF-7	PC3	HaCaT	MCF-7	PC3
**C_8_-LL-I**	5.69	10.55	8.07	1.42	0.76
**C_10_-LL-I**	7.54	11.97	10.10	1.34	0.84
**C_8_-LK6**	8.71	20.04	29.97	3.44	1.50
**C_8_-ATRA-1**	11.52	43.04	98.51	8.55	2.29
**C_10_-ATRA-1**	14.99	36.26	142.00	9.47	3.92

^1^ The selectivity index (SI) was calculated by dividing the IC_50_ value obtained against HaCaT by the IC_50_ value for MCF-7 and PC3 cancer cells.

**Table 3:** α-Helix percentage determined from the CD spectra.

Compounds	POPC	θ_222_/θ_208_	POPG	θ_222_/θ_208_
**LL-I**	20	0.33	37	1.00
**C_8_-LL-I**	48	0.85	27	0.90
**C_10_-LL-I**	40	0.88	19	0.98
**LK6**	15	0.41	23	1.17
**C_8_-LK6**	46	0.82	24	1.00
**ATRA-1**	-	-	10	2.27
**C_8_-ATRA-1**	11	0.38	20	1.23
**C_10_-ATRA-1**	14	0.65	17	1.28
	***Staphylococcus aureus***		**θ_222_/θ_208_**	
**C_8_-LK6, OD_0.1_**	22		1.26	
**C_8_-LK6, OD_0.2_**	17		0.89	
**C_8_-LK6, OD_0.5_**	18		0.88	

**Table 4:** Thermodynamic parameters of lipopeptide binding to POPC and POPG LUVs.

Compounds	N	pK_ITC_	Δ*H* (kcal mol^−1^)	TΔ*S* (kcal mol^−1^)	Δ*G* (kcal mol^−1^)
**POPG**					
**C_8_-LL-I**	2.05 ± 0.18	3.98 ± 0.06	−10.9 ± 1.36	−3.10 ± 1.36	−7.80 ± 0.09
**C_10_-LL-I**	1.31 ± 0.58	6.02 ± 1.09	−0.01 ± 0.03	10.60 ± 1.49	−10.60 ± 1.49
	0.13 ± 1.53	4.69 ± 0.54	−3.11 ± 38.3	5.66 ± 38.3	−8.77 ± 0.73
**C_8_-LK6**	1.02 ± 0.05	6.31 ± 0.34	0.97 ± 0.05	12.00 ± 0.46	−11.00 ± 0.46
**C_8_-ATRA-1**	2.84 ± 0.08	5.78 ± 0.27	0.27 ± 0.01	10.5 ± 0.36	−10.30 ± 0.36
**C_10_-ATRA-1**	3.16 ± 0.05	7.06 ± 0.61	0.22 ± 0.01	12.20 ± 0.83	−10.20 ± 0.83
**POPC**					
**C_10_-LL-I**	0.52 ± 0.34	4.42 ± 0.24	−1.16 ± 0.85	7.24 ± 0.91	−8.4 ± 0.34

## Data Availability

Data are contained within the article.

## Supplementary Materials

The supporting information can be downloaded at: https://www.mdpi.com/article/10.3390/ijms24043951/s1. Reference [65] is cited in the supplementary materials.

## Abbreviations

C16	Palmitic acid
C14	Myristic acid
C12	Lauric acid
C10	Capric acid
C8	Caprylic acid
CDL2	Cardiolipin 2
CG MD	Coarse-grained molecular dynamics
DPPC	1,2-Dipalmitoyl-*sn*-glycero-3-phosphocholine
DPPG	1,2-Dipalmitoyl-*sn*-glycero-3-[phospho-rac-(1-glycerol)]
LUV	Large unilamellar vesicle
LysPG	Lysyl-phosphatidylglycerol
MLV	Multilayer vesicle
POPC	1-Palmitoyl-2-oleoyl-*sn*-glycero-3-phosphocholine
POPE	1-Palmitoyl-2-oleoyl-*sn*-glycero-3-phosphatidylethanolamine
POPG	1-Palmitoyl-2-oleoyl-*sn*-glycero-3-[phospho-rac-(1-glycerol)]

## References

[1] World Health Organization (WHO) Antimicrobial Resistance, 17 November 2021. https://www.who.int/news-room/fact-sheets/detail/antimicrobial-resistance.

[2] Ventola C.L. (2015). The Antibiotic Resistance Crisis: Part 1—Causes and threats. Pharm. Ther..

[3] Reygaert W.C. (2018). An overview of the antimicrobial resistance mechanisms of bacteria. AIMS Microbiol..

[4] Rima M., Rima M., Fajloun Z., Sabatier J.M., Bechinger B., Naas T. (2021). Antimicrobial peptides: A potent alternative to antiviotics. Antibiotics.

[5] Haney E.F., Straus S.K., Hancock R.E.W. (2019). Reassessing the Host Defense Peptide Landscape. Front. Chem..

[6] Andersson D.I., Hughes D., Kubicek-Sutherland J.Z. (2016). Mechanisms and consequences of bacterial resistance to antimicrobial peptides. Drug Resist. Updat..

[7] Kumar P., Kizhakkedathu J.N., Straus S.K. (2018). Antimicrobial Peptides: Diversity, Mechanism of Action and Strategies to Improve the Activity and Biocompatibility In Vivo. Biomolecules.

[8] Mookherjee N., Anderson M.A., Haagsman H.P., Davidson D.J. (2020). Antimicrobial host defence peptides: Functions and clinical potential. Nat. Rev. Drug Discov..

[9] Gagnon M.C., Strandberg E., Grau-Campistany A., Wadhwani P., Reichert J., Brück J., Rabanal F., Auger M., Paquin J.F., Urlich A.S. (2017). Influence of the length and charge on the activity of α-helical amphipatic antimicrobial peptides. Biochemistry.

[10] Tossi A., Sandri L., Giangaspero A. (2000). Amphipathic, α-helical antimicrobial peptides. Biopolymers.

[11] Kosikowska P., Pikula M., Langa P., Trzonkowski P., Obuchowski M., Lesner A. (2015). Synthesis and Evaluation of Biological Activity of Antimicrobial—Pro-Proliferative Peptide Conjugates. PLoS ONE.

[12] Kang S.-J., Nam S.H., Lee B.-J. (2022). Engineering Approaches for the Development of Antimicrobial Peptide-Based Antibiotics. Antibiotics.

[13] Gong H., Sani M.A., Hu X., Fa K., Hart J.W., Liao M., Hollowell P., Carter J., Clifton L.A., Campana M. (2020). How do self-assembling antimicrobial lipopeptides kill bacteria?. ACS Appl. Mater. Interfaces.

[14] Boman H.G., Agerberth B., Boman A. (1993). Mechanism of action on Escherichia coli of cecropin P1 and PR-39, two antibacterial peptides from pig intestine. Infect. Immun..

[15] Chileveru H.R., Lim S.A., Chairatana P., Wommack A.J., Chiang I.-L., Nolan E.M. (2015). Visualizing Attack of *Escherichia coli* by the Antimicrobial Peptide Human Defensin. Biochemistry.

[16] Nishikata M., Kanehira T., Oh H., Tani H., Tazaki M., Kuboki Y. (1992). Salivary histatin ad an inhibitor of a protease produced by the oral bacterium Bacteroides gingivalis. Biochem. Biophys. Res. Commun..

[17] Lai Y., Gallo R.L. (2009). AMPed up immunity: How antimicrobial peptides have multiple roles in immune defense. Trends Immunol..

[18] Amerikova M., El-Tibi I.P., Maslarska V., Bozhanov S., Tachkov K. (2019). Antimicrobial activity, mechanism of action and methods for stabilisation of defensins as new therapeutic agents. Biotechnol. Biotechnol. Equip..

[19] Zhang L., Bulaj G. (2012). Converting peptides into drug leads by lipidation. Curr. Med. Chem..

[20] Kowalczyk R., Harris P.W.R., Williams G.M., Yang S.H., Brimble M.A. (2017). Peptide Lipidation—A Synthetic Strategy to Afford Peptide Based Therapeutics. In: Sunna, A., Care, A., Bergquist, P. (eds) Peptides and Peptide-based Biomaterials and their Biomedical Applications. Adv. Exp. Med. Biol..

[21] Drayton M., Kizhakkedathu J.N., Straus S.K. (2020). Towards Robust Delivery of Antimicrobial Peptides to Combat Bacterial Resistance. Molecules.

[22] Chen B., Sun Y., Niu J., Jarugumilli G.K., Wu X. (2018). Protein lipidation in cell signalling and diseases: Function, regulation, and therapeutic opportunities. Cell Chem. Biol..

[23] Vaezi Z., Bortolotti A., Luca V., Perilli G., Mangoni M.L., Khosravi-Far R., Bobone S., Stella L. (2020). Aggregation determines the selectivity of membrane-active anticancer and sntimicrobial peptides: The case of killerFLIP. Biochim. Biophys. Acta Biomembr..

[24] Kamysz E., Sikorska E., Jaśkiewicz M., Bauer M., Neubauer D., Bartoszewska S., Barańska-Rybak W., Kamysz W. (2020). Lipidated analogs of the LL-37-derived peptide fragment KR-12—Structural analysis, surface-active properties and antimicrobial activity. Int. J. Mol. Sci..

[25] Čeřovský V., Buděšínský M., Hovorka O., Cvačka J., Voburka Z., Slaninová J., Borovičková L., Fučík V., Bednárová L., Votruba I. (2009). Lasioglossins: Three Novel Antimicrobial Peptides from the Venom of the Eusocial Bee*Lasioglossum laticeps*(Hymenoptera: Halictidae). Chembiochem.

[26] Oliva R., Mukherjee S.K., Fetahaj Z., Moebitz S., Winter R., Oliva R. (2020). Perturbation of liquid droplets of P-granule protein LAF-1 by the antimicrobial peptide LL-III. Chem. Commun..

[27] Shang D., Li X., Sun Y., Wang C., Sun L., Wei S., Gou M. (2012). Design of Potent, Non-Toxic Antimicrobial Agents Based upon the Structure of the Frog Skin Peptide, Temporin-1CEb from Chinese Brown Frog, Rana chensinensis. Chem. Biol. Drug Des..

[28] de Latour F.A., Amer L.S., Papanstasiou E.A., Bishop B.M., van Hoek M.L. (2010). Antimicrobial activity of the Naja atra cathelicidin and related small peptides. Biochem. Biophys. Res. Commun..

[29] Meena K.R., Kanwar S.S. (2015). Lipopeptides as the Antifungal and Antibacterial Agents: Applications in Food Safety and Therapeutics. BioMed. Res. Int..

[30] Jiang Z., Kullberg B.J., Van Der Lee H., Vasil A.I., Hale J.D., Mant C.T., Hancock R.E.W., Vasil M.L., Netea M.G., Hodges R.S. (2008). Effects of Hydrophobicity on the Antifungal Activity of α-Helical Antimicrobial Peptides. Chem. Biol. Drug Des..

[31] Vieira D.B., Carmona-Ribeiro A.M. (2006). Cationic lipids and surfactants as antifungal agents: Mode of action. J. Antimicrob. Chemother..

[32] Zai Y., Xi X., Ye Z., Ma C., Zhou M., Chen X., Siu S., Chen T., Wang L., Kwok H. (2021). Aggregation and Its Influence on the Bioactivities of a Novel Antimicrobial Peptide, Temporin-PF, and Its Analogues. Int. J. Mol. Sci..

[33] Manzo G., Scorciapino M.A., Wadhwani P., Bürck J., Montaldo N.P., Pintus M., Sanna R., Casu M., Giuliani A., Pirri G. (2015). Enhanced Amphiphilic Profile of a Short β-Stranded Peptide Improves Its Antimicrobial Activity. PLoS ONE.

[34] Zhou N.E., Kay C.M., Hodges R.S. (1992). Synthetic model proteins. Positional effects of interchain hydrophobic interactions on stability of two-stranded alpha-helical coiled-coils. J. Biol. Chem..

[35] Malik L., Nygaard J., Christensen N.J., Straicher W.W., Thulstrup P.W., Arleth L., Jensen K.J. (2013). Self-assembly of designed coiled coil peptides studied by small-angle X-ray scattering and analytical ultracentrifugation. J. Pept. Sci..

[36] Crooks P.O., Rao T., Mason J.M. (2011). Truncation, randomization, and selection: Generation of a reduced length c-Jun antagonist that retains high interaction stability. J. Biol. Chem..

[37] Hoernke M., Schwieger C., Kerth A., Blume M. (2012). Binding of cationic pentapeptides with modified side chain lengths to negatively charged lipid membranes: Complex interplay of electrostatic and hydrophobic interactions. Biochim. Biophys. Acta (BBA)—Biomembr..

[38] Kodati V.R., El-Jastimi R., Lafleur M. (1994). Contribution of the Intermolecular Coupling and Librotorsional Mobility in the Methylene Stretching Modes in the Infrared Spectra of Acyl Chains. J. Phys. Chem..

[39] Arias J.M., Picot R.A.C., Tuttolomondo M.E., Ben Altabef A., Díaz S.B. (2020). Interaction of *N*-acetylcysteine with DPPC liposomes at different pH: A physicochemical study. New J. Chem..

[40] Crowe L.M., Crowe J.H. (1991). Solution effects on the thermotropic phase transition of unilamellar liposomes. Biochim. Biophys. Acta (BBA)—Biomembr..

[41] Chen C., Tripp C.P. (2012). A comparison of the behavior of cholesterol, 7-dehydrocholesterol and ergosterol in phospholipid membranes. Biochim. Biophys. Acta (BBA)—Biomembr..

[42] Małuch I., Stachurski O., Kosikowska-Adamus P., Makowska M., Bauer M., Wyrzykowski D., Hać A., Kamysz W., Deptuła M., Pikuła M. (2020). Double-Headed Cationic Lipopeptides: An Emerging Class of Antimicrobials. Int. J. Mol. Sci..

[43] Mondal S., Gazit E. (2016). The Self-Assembly of Helical Peptide Building Blocks. Chemnanomat.

[44] Mendes A.C., Baran E.T., Reis R.L., Azevedo H.S. (2013). Self-assembly in nature: Using the principles of nature to create complex nanobiomaterials. WIREs Nanomed. Nanobiotechnol..

[45] DeGrado W.F., Gratkowski H., Lear J.D. (2003). How do helix-helix interactions help determine the folds of membrane proteins? Perspectives from the study of homo-oligomeric helical bundles. Protein Sci..

[46] Heifetz A., Morao I., Babu M.M., James T., Southey M.W.Y., Fedorov D.G., Aldeghi M., Bodkin M.J., Townsend-Nicholson A. (2020). Characterizing Interhelical Interactions of G-Protein Coupled Receptors with the Fragment Molecular Orbital Method. J. Chem. Theory Comput..

[47] Marchesan S., Vargiu A.V., Styan K.E. (2015). The Phe-Phe Motif for Peptide Self-Assembly in Nanomedicine. Molecules.

[48] Wayne P.A. (2008). CLSI. Methods for Dilution Antimicrobial Susceptibility Test for Bacteria that Grow Aerobically.

[49] Wayne P.A. (2008). CLSI. Reference Method for Broth Dilution Antifungal Susceptibility Testing of Yeast.

[50] Kamysz E., Smolarczyk R., Cichoń T., Jarosz-Biej M., Sikorska E., Sobocińska M., Jaśkiewicz M., Kamysz W. (2016). Antitumor activity of opiorphin, sialorphin and their conjugated with a peptide klaklakklaklak. J. Pept. Sci..

[51] Sikorska E., Dawgul M., Greber K., Iłowska E., Pogorzelska A., Kamysz W. (2014). Self-assembly and interactions of short antimicrobial cationic lipopeptides with membrane lipids: ITC, FTIR and molecular dynamics studies. Biochim. Biophys. Acta (BBA)—Biomembr..

[52] Neubauer D., Jaśkiewicz M., Sikorska E., Bauer S.B.M., Kapusta M., Narajczyk M., Kamysz W. (2020). Effect of Disulfide Cyclization of Ultrashort Cationic Lipopeptides on Antimicrobial Activity and Cytotoxicity. Int. J. Mol. Sci..

[53] Case D.A., Ben-Shalom I.Y., Brozell S.R., Cerutti D.S., Cheatham T.E., Cruzeiro V.W.D., Darden T.A., Duke R.E., Ghoreishi D., Gilson M.K. (2018). Amber 18. 2008.

[54] Schmidt M.W., Baldridge K.K., Boatz J.A., Elbert S.T., Gordon M.S., Jensen J.H., Koseki S., Matsunaga N., Nguyen K.A., Su S. (1993). General atomic and molecular electronic structure system. J. Comput. Chem..

[55] Bayly C.I., Cieplak P., Cornell W.D., Kollman P.A. (1993). A well-behaved electrostatic potential based method using charge restraints for deriving atomic charges: The RESP model. J. Phys. Chem..

[56] Martínez L., Andrade R., Birgin E.G., Martínez J.M. (2009). PACKMOL: A package for building initial configurations for molecular dynamics simulations. J. Comput. Chem..

[57] Hess B., Kutzner C., van der Spoel D., Lindahl E. (2008). GROMACS 4: Algorithms for Highly Efficient, Load-Balanced, and Scalable Molecular Simulation. J. Chem. Theory Comput..

[58] Humphrey W., Dalke A., Schulten K. (1996). VMD: Visual molecular dynamics. J. Mol. Graph..

[59] Periole X., Marrink S.J. (2013). The Martini coarse-grained force field. Methods Mol. Biol..

[60] Marrink S.J., Risselada H.J., Yefimov S., Tieleman D.P., de Vries A.H. (2007). The martini Force Field: Coarse Grained Model for Biomolecular Simulations. J. Phys. Chem. B.

[61] Piggot T.J., Holdbrook D.A., Khalid S. (2011). Electroporation of the *E. coli* and *S. Aureus* Membranes: Molecular Dynamics Simulations of Complex Bacterial Membranes. J. Phys. Chem. B.

[62] Winger M., Trzesniak D., Baron R., van Gunsteren W.F. (2009). On using a too large integration time step in molecular dynamics simulations of coarse-grained molecular models. Phys. Chem. Chem. Phys..

[63] Gapsys V., De Groot B.L., Briones R. (2013). Computational analysis of local membrane properties. J. Comput. Aided Mol. Des..

[64] Thota C.K., Mikolajczak D.J., Roth C., Koksch B. (2021). Enhancing Antimicrobial Peptide Potency through Multivalent Presentation on Coiled-Coil Nanofibrils. ACS Med. Chem. Lett..

[65] Eisenberg D. (1984). Three-dimensional structure of membrane and surface proteins. Ann. Rev. Biochem..

