# Supplementary material for: Lipidation of Naturally Occurring α-Helical Antimicrobial Peptides as a Promising Strategy for Drug Design
Source: Int J Mol Sci. 2023 Feb 16;24(4):3951. doi: 10.3390/ijms24043951 (PMC9959048; doi:10.3390/ijms24043951)
Supplement: Supplementary file 1 [file ijms-24-03951-s001.zip › ijms-2189249-supplementary.pdf]

Supplementary materials

Lipidation of Naturally Occurring α-Helical Antimicrobial Peptides as a Promising Strategy for Drug Design

Marta Makowska [1,*], Paulina Kosikowska-Adamus [1], Magdalena Zdrowowicz [2], Dariusz Wyrzykowski [3], Adam Prahl [1] and Emilia Sikorska [1,*]

[1] Department of Organic Chemistry, Faculty of Chemistry, University of Gdansk, Wita Stwosza 63, 80-308 Gdansk, Poland
[2] Department of Physical Chemistry, Faculty of Chemistry, University of Gdansk, Wita Stwosza 63, 80-308 Gdansk, Poland
[3] Department of General and Inorganic Chemistry, Faculty of Chemistry, University of Gdansk, Wita Stwosza 63, 80-308 Gdansk, Poland;
* Correspondence: marta.makowska@phdstud.ug.edu.pl (M.M.); emilia.sikorska@ug.edu.pl (E.S.)

Table S1. List of peptides described in this paper with characteristics of their biological properties.

Peptide	Sequence	Aaa	Net charge	Mean residue hydrophobicity[1]	Biological activity
**LL-I**	VNWKKVLGKIIKVAK-NH_2	15	+6	-0.117	Peptide isolated from the venom of the wild eusocial bee *L. laticeps* [1]. This peptide is built of 15 amino acid residues, half of which are hydrophobic, and the other half are polar - including five L-lysine residues [2]. In water and in the presence of small amounts of substances imitating bacterial cell membranes (TFE - trifluoroethanol or SDS, sodium dodecyl sulfate) LL-I remains disordered, while at higher concentrations of TFE or SDS, it adopts the α-helical structure [1]. Antimicrobial assays revealed that LL-I exhibits strong antimicrobial activity against both Gram-positive (*S. aureus*, *B. subtilis*) and Gram-negative (*E. coli*, *P. aeruginosa*) bacteria but the mechanism of its action remains unknown. Additionally, LL-I causes a cytotoxic effect on rat pheochromocytoma cells, and therefore this compound is considered as a peptide with potent anticancer properties.
**LK6**	IKKILSKIKKLLK-NH_2	13	+7	-0.165	One of two analogues of the naturally occurring temporin-1CEb isolated from the secretion covering the skin of the *Rana amurensis* frog. Cationic α-helical

					peptide with strong antimicrobial activity against Gram-positive (*S. aureus, B. cereus, S. lactis, E. faecalis, E. faecium*) and Gram-negative bacteria (*E. coli, P. aeruginosa, E. aerogenes, E. cloacae, K. pneumoniae*) and low haemolytic activity. The proposed mechanism of action is based on the depolarization of microorganisms' cell membranes [3].
**ATRA-1**	KRFKKFFKKLK-NH_2	11	+8	-0.549	The analogue is the initial 11-amino acid fragment of the NA-CATH peptide – cathelicidin presented in the Chinese cobra *Naja atra*. This peptide shows high antimicrobial activity against *E. coli* and the oral bacteria *A. actinomycetemcomitans*. Analysis by the SSPro predictor for secondary structure predicted that ATRA-1 had a significant helical character, which was confirmed experimentally. The CD experiment showed a helical nature of this analogue in 90 mM SDS. It has been proven that the combination of the antimicrobial activity of ATRA-1 with its low haemolysis can provide a promising basis for the design of new peptide antibiotics [4].

[1]Mean residue hydrophobicity was calculated on the basis of the Eisenberg consensus scale of hydrophobicity [5].

References:

1. Cerovský, V.; Budesínský, M.; Hovorka, O.; Cvacka, J.; Voburka, Z.; Slaninová, J.; Borovicková, L., Fucík, V.; Bednárová, L.; Votruba, I.; Straka, J. Lasioglossins: three novel antimicrobial peptides from the venom of the eusocial bee Lasioglossum laticeps (Hymenoptera: Halictidae). *Chembiochem.* **2009**, 10, 2089-99.
2. Oliva, R.; Mukherjee, S.K.; Fetahaj, Z.; Möbitz, S.; Winter, R. Perturbation of liquid droplets of P-granule protein LAF-1 by the antimicrobial peptide LL-III. *Chem. Commun.* **2020**, 56, 11577-11580.
3. Shang, D.; Li, X.; Sun, Y.; Wang, C.; Sun, L.; Wei, S.; et al. Design of Potent, Non-Toxic Antimicrobial Agents Based upon the Structure of the Frog Skin Peptide, Temporin-1CEb from Chinese Brown Frog, Rana chensinensis. *Chem. Biol. Drug Des.* **2012**, 79, 653-662.
4. de Latour, F.A.; Amer, L.S.; Papanstasiou, E.A.; Bishop, B.M.; van Hoek, M.L. Antimicrobial activity of the Naja atra cathelicidin and related small peptides. *Biochem. Biophys. Res. Commun.* **2010**, 396, 825-830.
5. Eisenberg, D. Three-dimensional structure of membrane and surface proteins. *Ann. Rev. Biochem.* **1984**, 53: 595-623.

Table S2. Analytical data of synthesized lipopeptides.

Compounds	Retention time [min]	Molecular mass [Da]	
		Calculated	MS-TOF $[M+H]^+$
**LL-I**	21.31[a]	1721.8	1723.1
**C_8-LL-I**	27.57[a]	1848.01	1848.57
**C_{10}-LL-I**	27.68[a]	1876.07	1877.16
**C_{12}-LL-I**	26.23[b]	1904.12	1905.3
**C_{14}-LL-I**	28.06[b]	1932.17	1933.34
**C_{16}-LL-I**	19.52[b]	1960.22	1961.4
**LK6**	18.34[b]	1552.1	1552.2
**C_8-LK6**	24.32[b]	1678.31	1678.29
**C_{10}-LK6**	26.22[b]	1706.37	1706.27
**C_{12}-LK6**	28.05[b]	1734.42	1734.33
**C_{14}-LK6**	19.59[c]	1762.47	1762.38
**C_{16}-LK6**	22.69[c]	1790.52	1790.37
**ATRA-1**	15.35[a]	1496.93	1496.99
**C_8-ATRA-1**	21.06[a]	1623.14	1623.06
**C_{10}-ATRA-1**	21.49[a]	1650.2	1651.08
**C_{12}-ATRA-1**	24.23[a]	1679.25	1679.32
**C_{14}-ATRA-1**	26.11[a]	1707.3	1707.21
**C_{16}-ATRA-1**	27.61[a]	1735.35	1735.26

[a] linear gradient 1-80% of B in A for 30 min, [b] linear gradient 15-90% of B in A for 30 min, [c] 50-100% of B in A for 30 min, flow rate 1 mL/min, RP-HPLC Shimadzu system, column: Jupiter 4 μm Proteo, 90 Å, 250 x 4.60 mm, A: 0.1% solution of TFA in water, B: 80% solution of acetonitrile in A.

Table S3. Composition of the bilayer systems.

Model	Lipid types		Ion types		Peptide	Water	System size [x,y,z; Å]	Simulation time [μs]
	**Outer**	**Inner**	**Na^+**	**Cl^-**				
**LB (lipid bilayer)**	261 DPPG<br>174 Lys-PG<br>48 CDL2	261 DPPG<br>174 Lys-PG<br>48 CDL2	1019	653	-	56348	170,170,250	10.0
**C_8-LK6**	261 DPPG<br>174 Lys-PG<br>48 CDL2	261 DPPG<br>174 Lys-PG<br>48 CDL2	1019	953	50	55476	176,176,270	10.0
**C_8-LL-I**	261 DPPG<br>174 Lys-PG<br>48 CDL2	261 DPPG<br>174 Lys-PG<br>48 CDL2	1019	903	50	55470	176,176,270	10.0
**C_{10}-ATRA-1**	261 DPPG<br>174 Lys-PG<br>48 CDL2	261 DPPG<br>174 Lys-PG<br>48 CDL2	1019	1003	50	55441	176,176,270	10.0

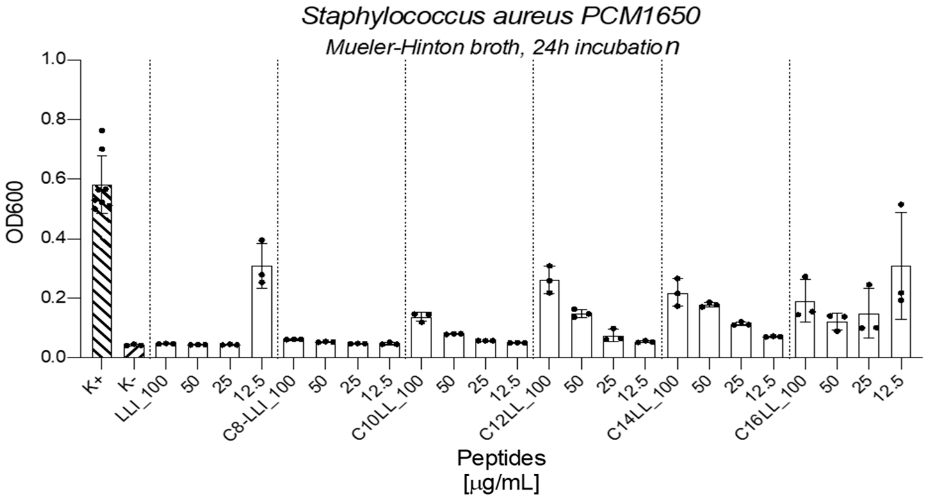

Figure S1. Effect of the lipopeptide concentration on the optical density at a wavelength of 600 nm. K+ and K- correspond to positive and negative controls.

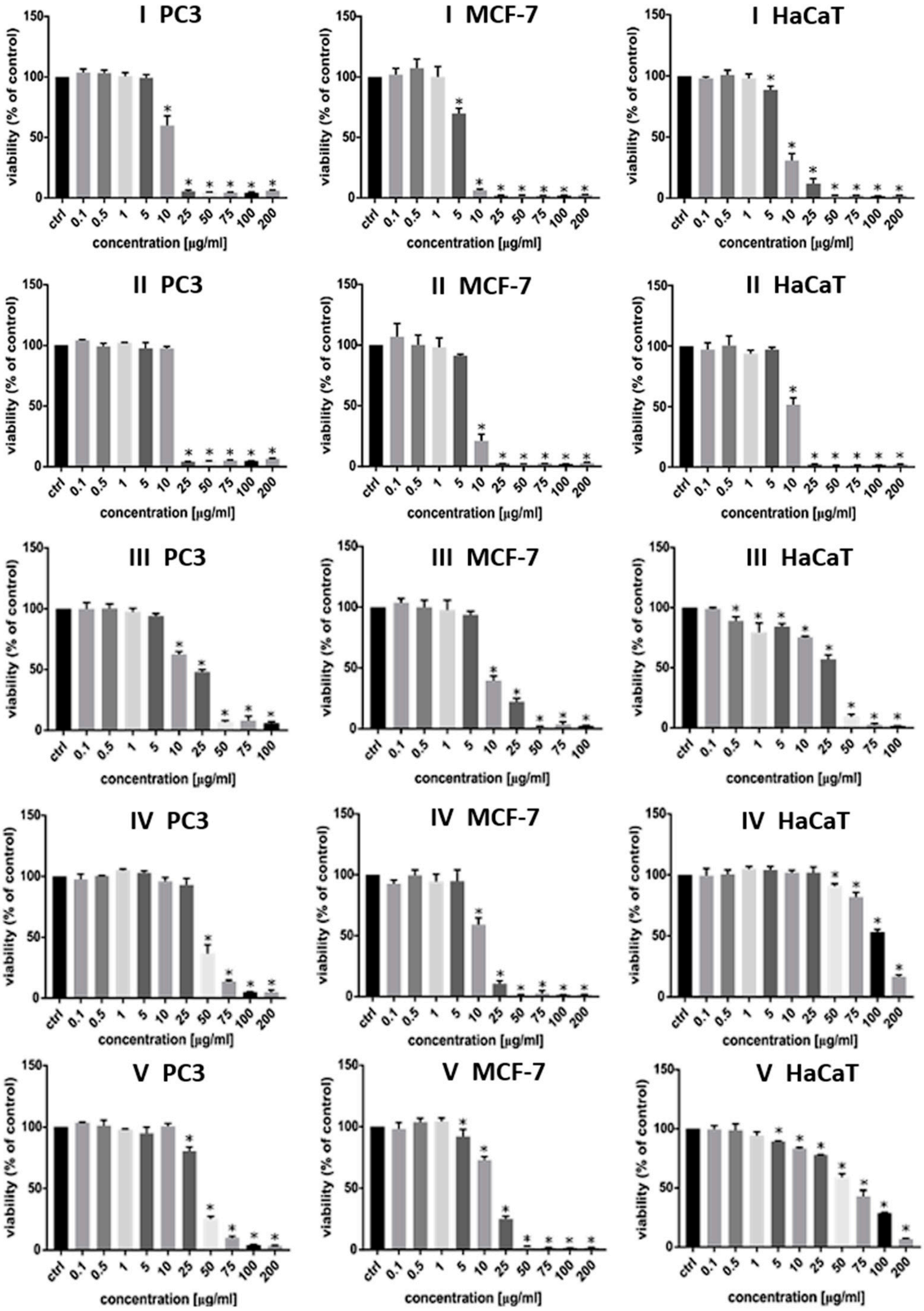

Figure S2. The viability of PC3, MCF-7 and HaCaT cells after 48 h treatment with tested compounds. Results are shown as the mean ± standard deviation (SD) of three independent experiments performed in triplicate. *A statistically significant difference is present between treated cultures compared with the control (untreated culture), $p < 0.05$. (**I** – C_8-LL-I, **II** – C_{10}-LL-I, **III** – C_8-LK6, **IV** – C_8-ATRA-1, **V** – C_{10}-ATRA-1).

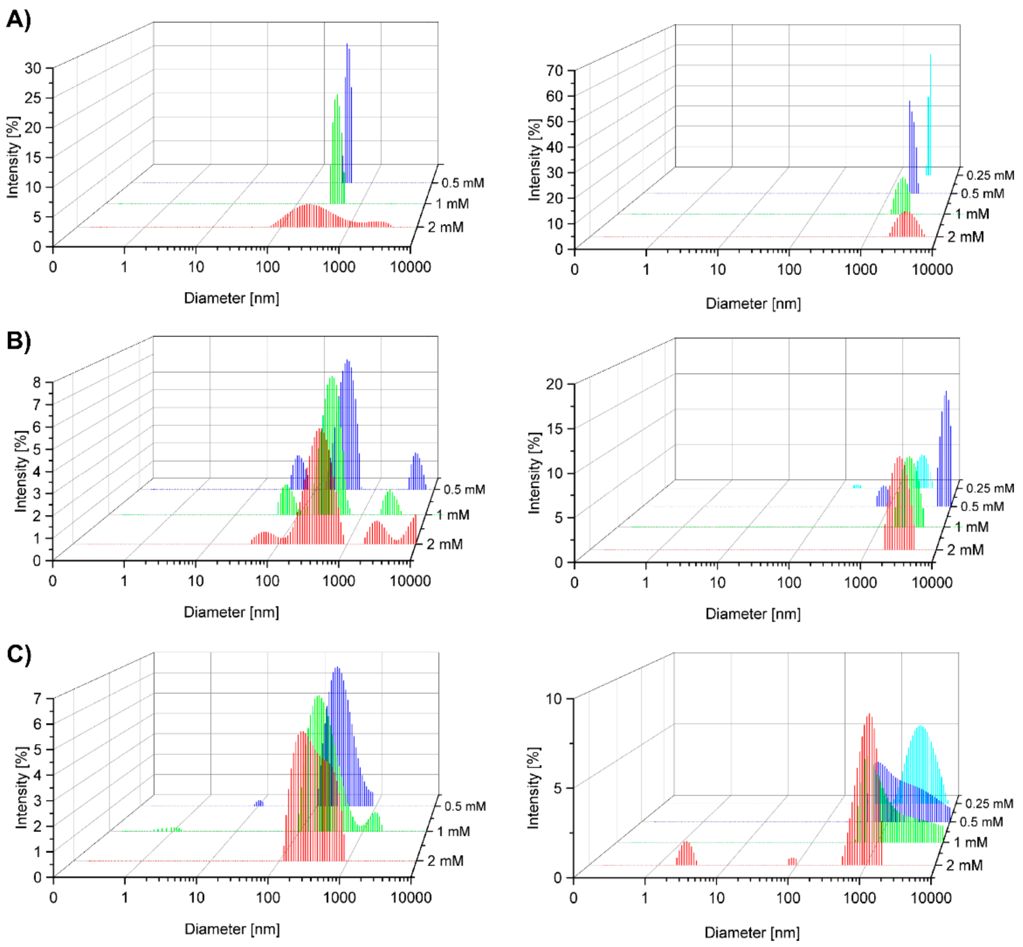

Figure S3. Dynamic light scattering distributions obtained in water (left panel) and PBS (right panel) for C_8-LL-I (**A**), C_8-LK6 (**B**) and C_{10}-ATRA-1 (**C**) at various peptide concentrations.

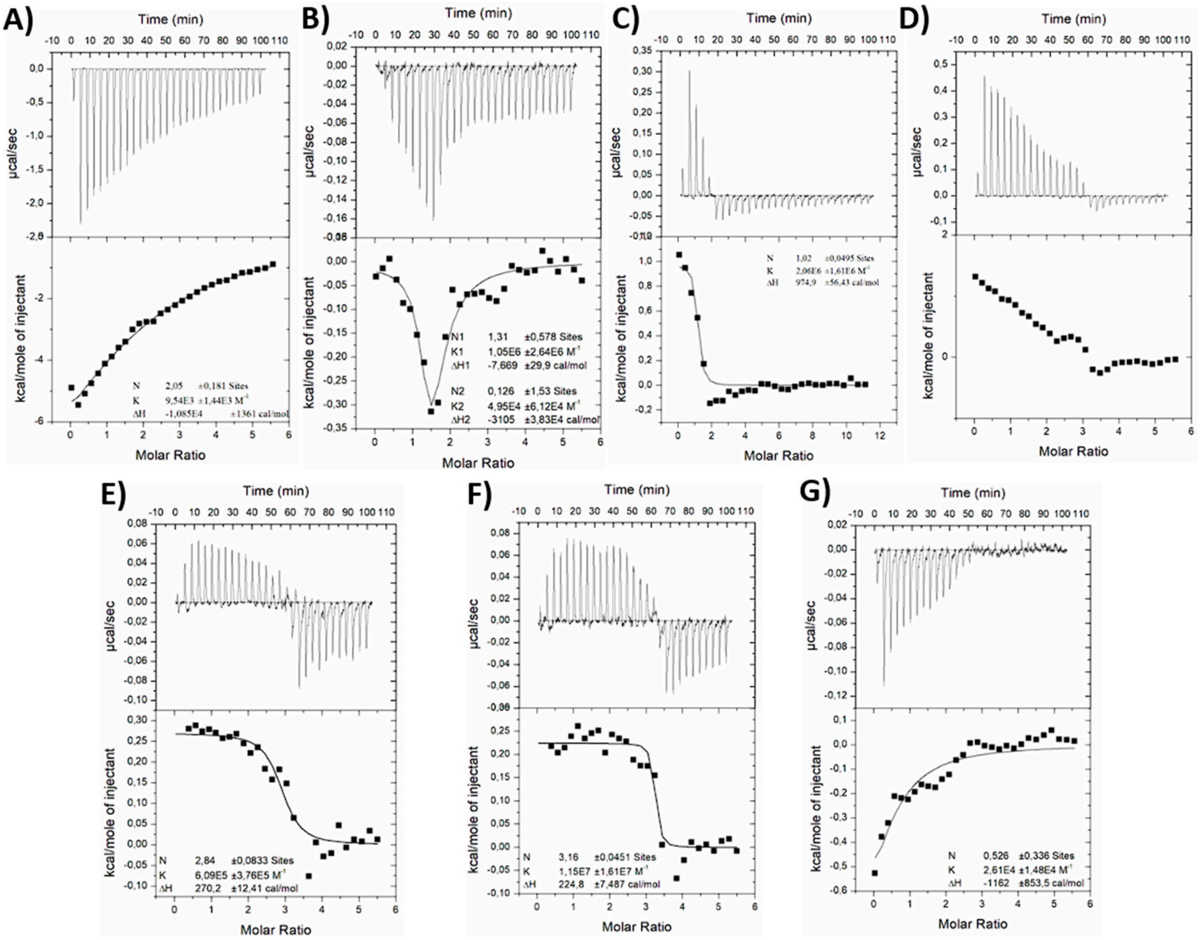

Figure S4. Isothermal titration at 298 K of (**A**) 1.3 mM POPG to 0.05 mM C_8-LL-I, (**B**) 1.8 mM POPG to 0.07 mM C_{10}-LL-I, (**C**) 1.3 mM POPG to 0.025 mM C_8-LK6, (**D**) 1.3 mM POPG to 0.05 mM C_8-LK6, (**E**) 1.8 mM POPG to 0.07 mM C_8-ATRA-1, (**F**) 1.8 mM POPG to 0.07 mM C_{10}-ATRA-1, and (**G**) 1.3 mM POPC to 0.05 mM C_{10}-LL-I.

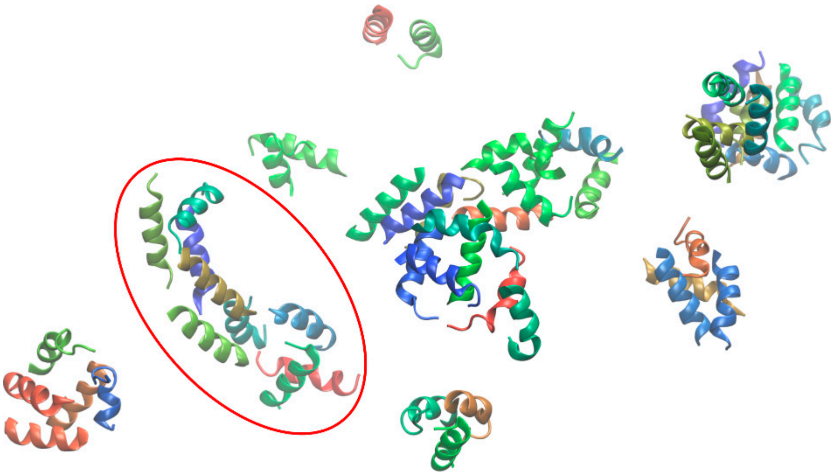

Figure S5. The final snapshot from the 500 ns full-atom simulations of C_8-LL-I self-assembly in solution. The structures are represented as a ribbon model. The red line indicates the largest oligomer (9-mer)

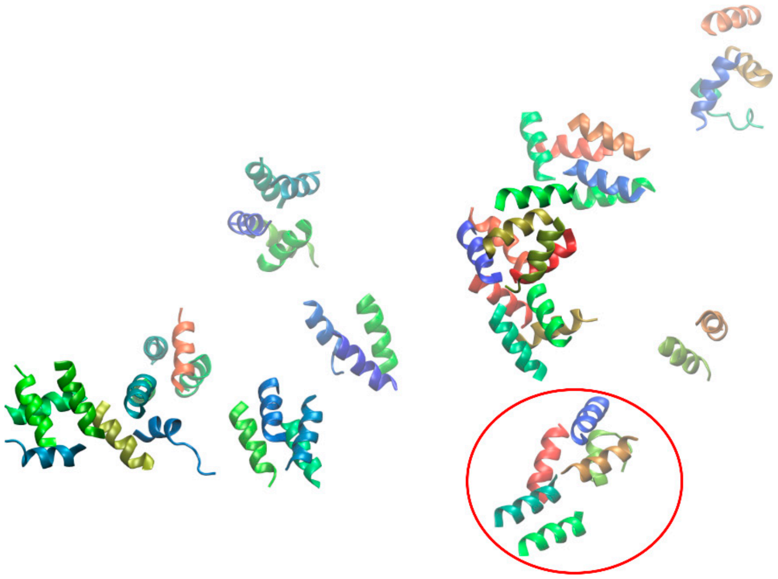

Figure S6. The final snapshot from the 500 ns full-atom simulations of C_8-LK6 self-assembly in solution. The structures are represented as a ribbon model. The red line indicates the largest oligomer (6-mer)

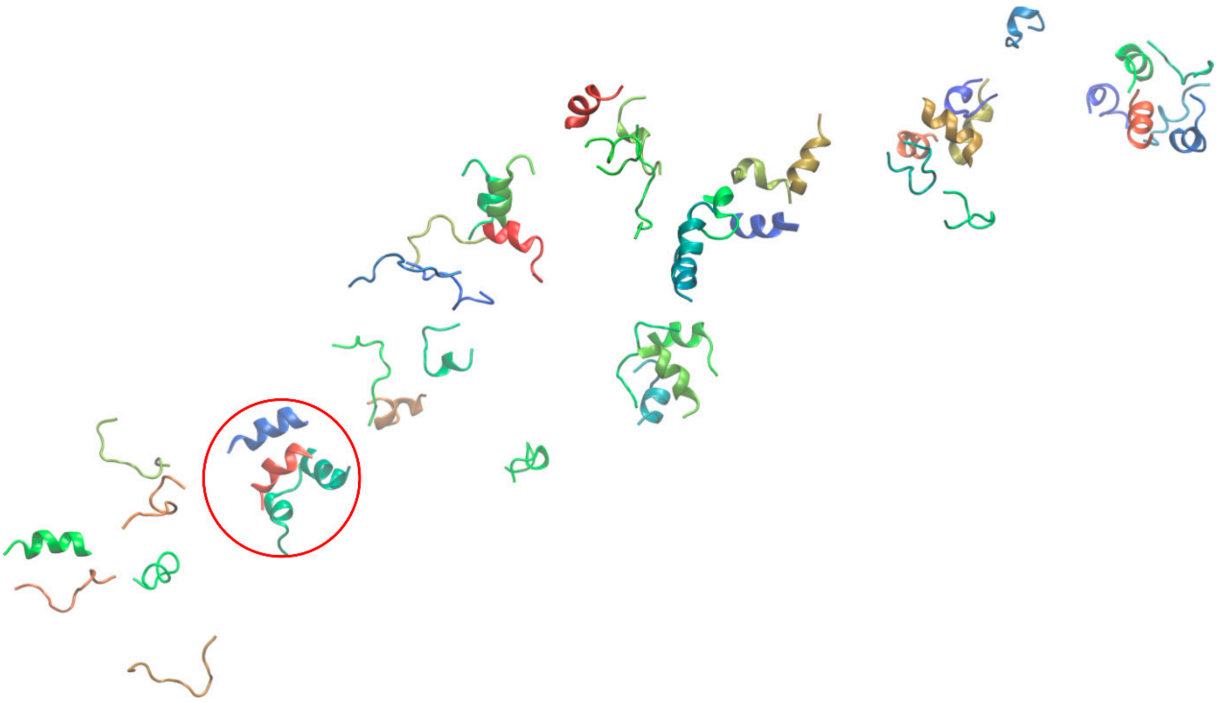

Figure S7. The final snapshot from the 500 ns full-atom simulations of C_{10}-ATRA-1 self-assembly in solution. The structures are represented as a ribbon model. The red line indicates the largest oligomer (4-mer)

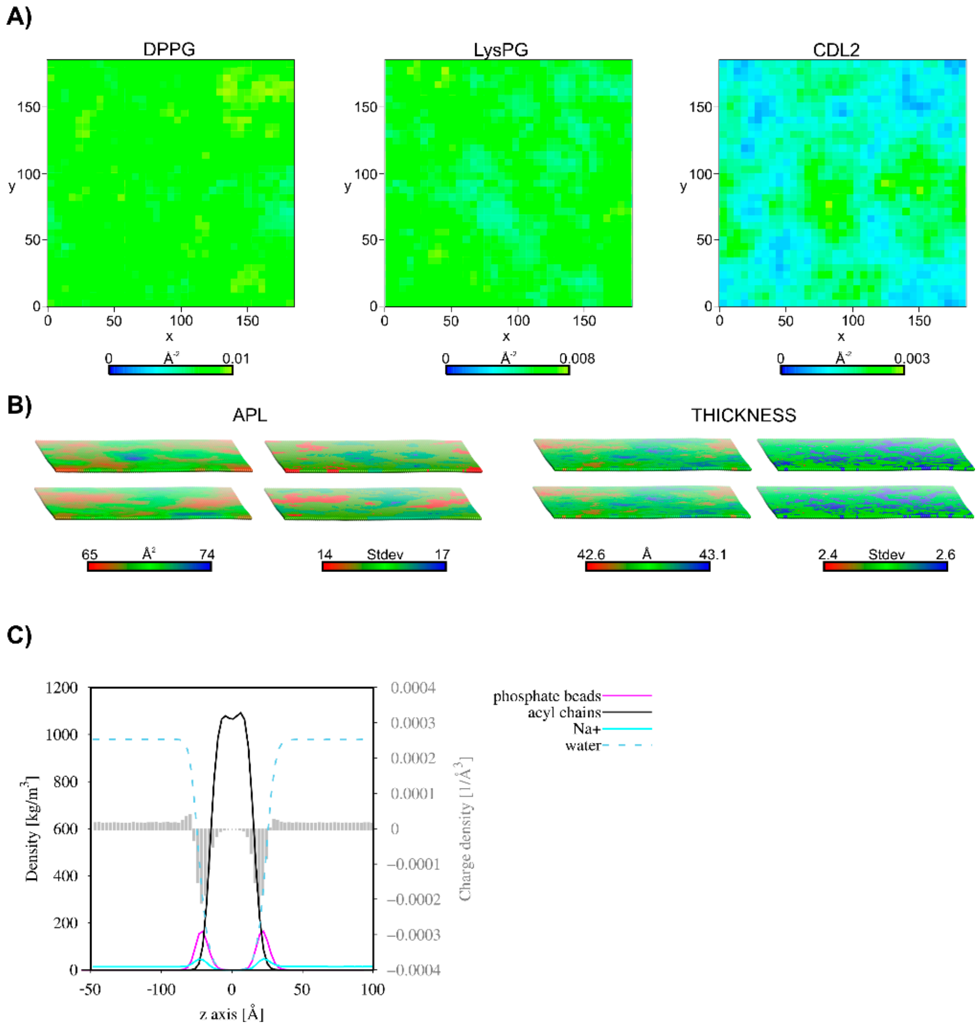

Figure S8. (A) 2D density map of the individual lipids in the upper leaflet of the DPPG/LysPG/CDL2 membrane. A grid spacing was set to 5 Å. **(B)** Local area per lipid (APL), thickness of the membrane and their standard deviations. **(C)** Partial density and charge density profiles.

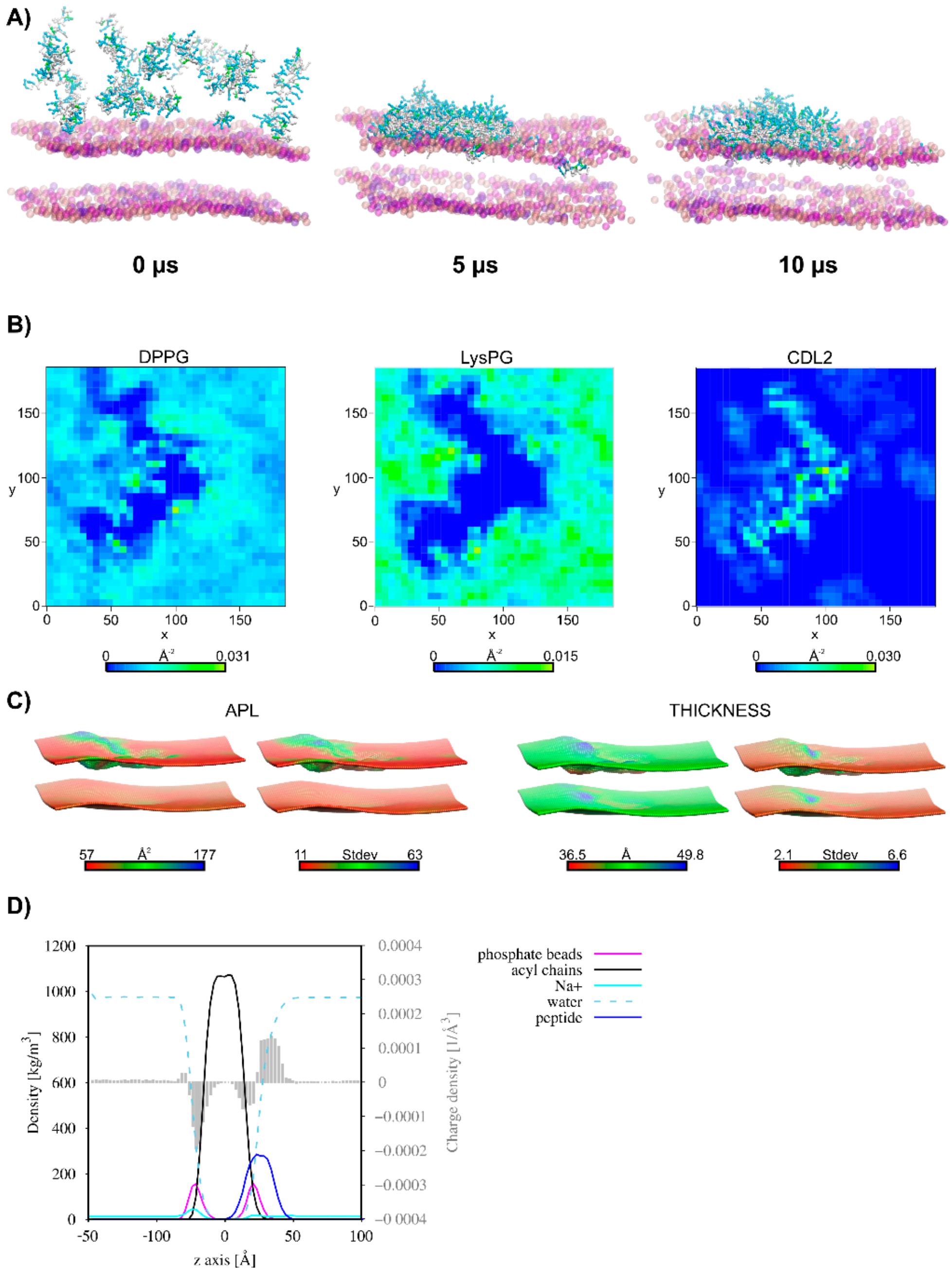

Figure S9. (A) Representative snapshots from the DPPG/LysPG/CDL2 binding CG MD simulations for C_8-LL-I. For clarity, only phosphate beads of the membrane have been displayed. The DPPG, LysPG and CDL2 phosphate groups are indicated in pink, magenta and violet, respectively. Octanoyl tails and nonpolar amino acids are in white, positively charged residues are in cyan and uncharged polar residues are in green. **(B)** 2D density map of the individual lipids in the upper leaflet of the DPPG/LysPG/CDL2 membrane in the system containing C_8-LL-I. A grid spacing was set to 5 Å. **(C)** Local area per lipid (APL), thickness of the membrane and their standard deviations (side view). **(D)** Partial density and charge density profiles.

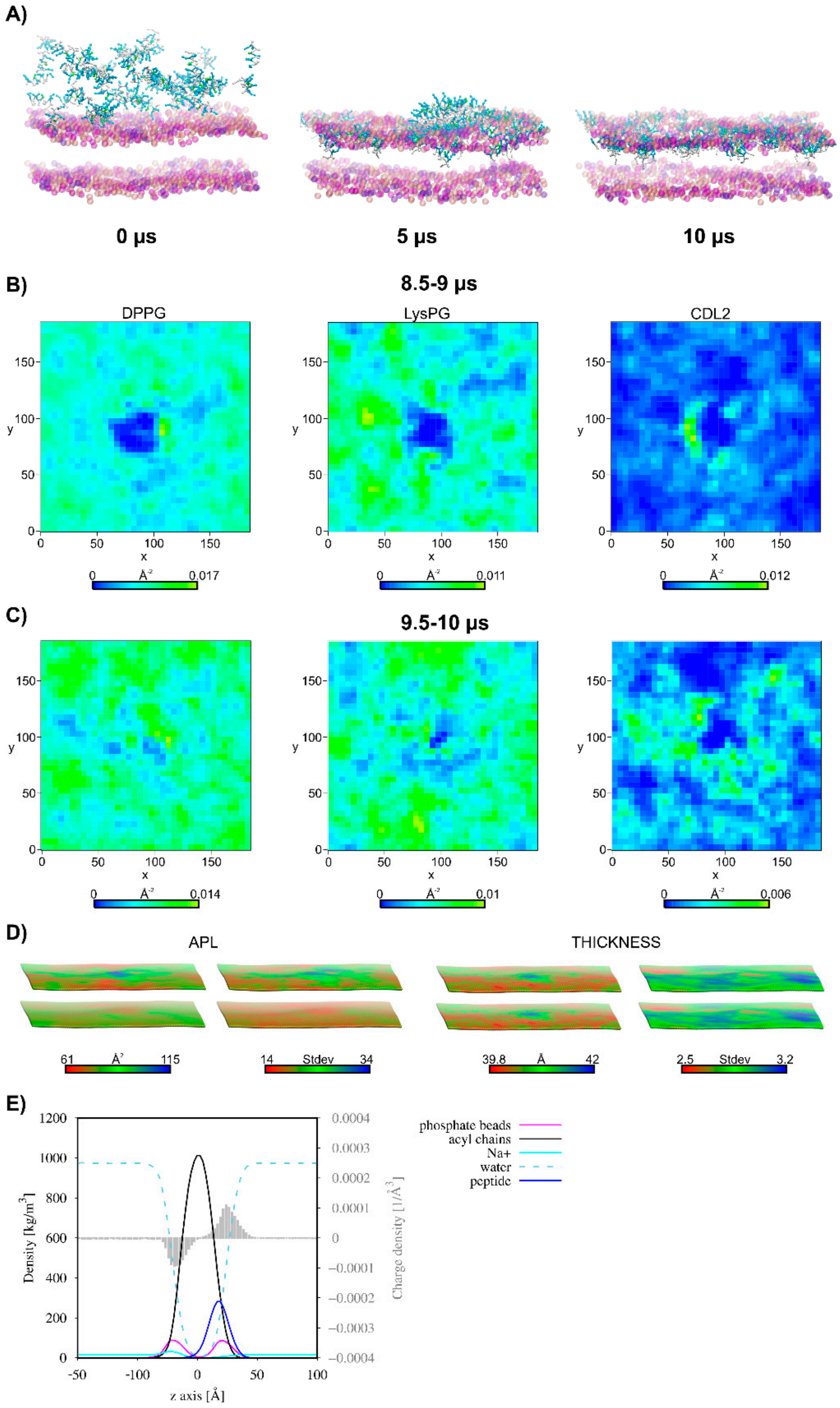

Figure S10. (A,B) 2D density map of the individual lipids in the upper leaflet of the DPPG/LysPG/CDL2 membrane in the system containing C8-LK6 averaged over two different CG MD periods. A grid spacing was set to 5 Å. **(C)** Local area per lipid (APL), thickness of the membrane and their standard deviations (side view). **(D)** Partial density and charge density profiles

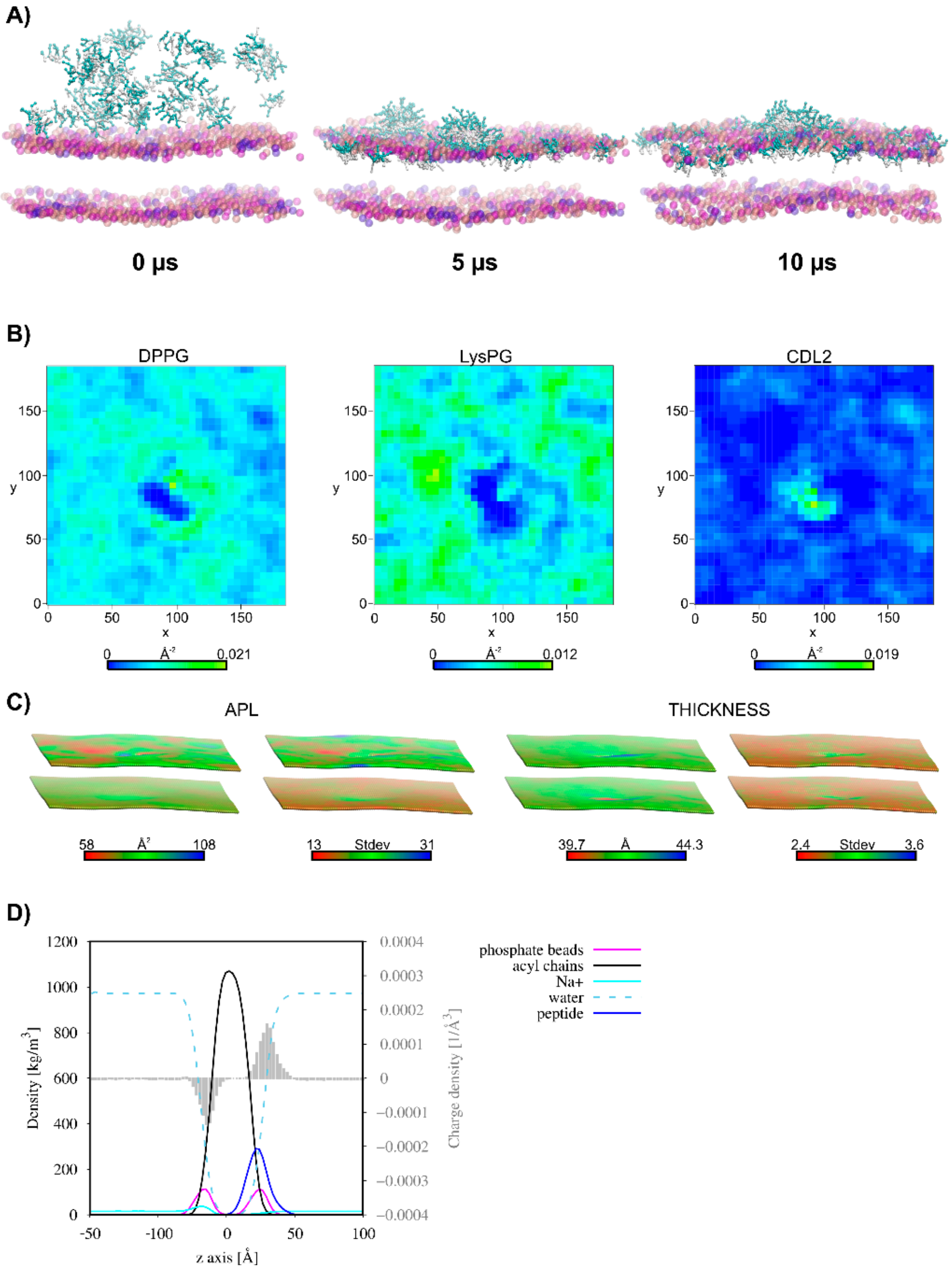

Figure S11. (A) Representative snapshots from the DPPG/LysPG/CDL2 binding CG MD simulations for C_{10}-ATRA-1. For clarity, only phosphate beads of the membrane have been displayed. The DPPG, LysPG and CDL2 phosphate groups are indicated in pink, magenta and violet, respectively. Decanoyl tails and nonpolar amino acids are in white, while positively charged residues are in cyan. **(B)** 2D density map of the individual lipids in the upper leaflet of the DPPG/LysPG/CDL2 membrane in the system containing C_{10}-ATRA-1. A grid spacing was set to 5 Å. **(C)** Local area per lipid (APL), thickness of the membrane and their standard deviations (side view). **(D)** Partial density and charge density profiles
